# Two Populations of Mites (*Tyrophagus putrescentiae*) Differ in Response to Feeding on Feces-Containing Diets

**DOI:** 10.3389/fmicb.2018.02590

**Published:** 2018-10-30

**Authors:** Jan Hubert, Marta Nesvorna, Bruno Sopko, Jaroslav Smrz, Pavel Klimov, Tomas Erban

**Affiliations:** ^1^Divison of Crop Protection and Plant Health, Crop Research Institute, Prague, Czechia; ^2^Department of Medical Chemistry and Clinical Biochemistry, Second Faculty of Medicine, Motol University Hospital, Charles University, Prague, Czechia; ^3^Department of Zoology, Faculty of Science, Charles University, Prague, Czechia; ^4^Department of Ecology and Evolutionary Biology, University of Michigan, Ann Arbor, MI, United States; ^5^Institute of Biology, University of Tyumen, Tyumen, Russia

**Keywords:** *Bartonella*, feeding, feces, transmission, soil, fungi, bacteria, diets

## Abstract

**Background:** *Tyrophagus putrescentiae* is a ubiquitous mite species in soil, stored products and house dust and infests food and causes allergies in people. *T. putrescentiae* populations harbor different bacterial communities, including intracellular symbionts and gut bacteria. The spread of microorganisms via the fecal pellets of *T. putrescentiae* is a possibility that has not been studied in detail but may be an important means by which gut bacteria colonize subsequent generations of mites. Feces in soil may be a vector for the spread of microorganisms.

**Methods:** Extracts from used mite culture medium (i.e., residual food, mite feces, and dead mite bodies) were used as a source of feces-inhabiting microorganisms as food for the mites. Two *T. putrescentiae* populations (L and P) were used for experiments, and they hosted the intracellular bacteria *Cardinium* and *Wolbachia*, respectively. The effects of the fecal fraction on respiration in a mite microcosm, mite nutrient contents, population growth and microbiome composition were evaluated.

**Results:** Feces from the P population comprised more than 90% *Bartonella*-like sequences. Feces from the L population feces hosted *Staphylococcus, Virgibacillus, Brevibacterium, Enterobacteriaceae*, and *Bacillus*. The mites from the P population, but not the L population, exhibited increased bacterial respiration in the microcosms in comparison to no-mite controls. Both L- and P-feces extracts had an inhibitory effect on the respiration of the microcosms, indicating antagonistic interactions within feces-associated bacteria. The mite microbiomes were resistant to the acquisition of new bacterial species from the feces, but their bacterial profiles were affected. Feeding of P mites on P-feces-enriched diets resulted in an increase in *Bartonella* abundance from 6 to 20% of the total bacterial sequences and a decrease in *Bacillus* abundance. The population growth was fivefold accelerated on P-feces extracts in comparison to the control.

**Conclusion:** The mite microbiome, to a certain extent, resists the acquisition of new bacteria when mites are fed on feces of the same species. However, a *Bartonella-*like bacteria-feces-enriched diet seems to be beneficial for mite populations with symbiotic *Bartonella*-like bacteria. Coprophagy on the feces of its own population may be a mechanism of bacterial acquisition in *T. putrescentiae*.

## Introduction

Bacterial symbionts can directly or indirectly affect the interaction of the host populations with other species within a community (Ferrari and Vavre, 2011). Many arthropod species are infected by more than one symbiont (Ferrari and Vavre, 2011). For example, spider mite (*Tetranychus phaselus*) populations can host multiple simultaneous infections by *Cardinium* and two distinct lineages of *Wolbachia* (Zhao et al., 2013). This complexity may generate a meta-community structure of host-associated microbiota. Members of stored food astigmatid mites (Acari: Astigmata) have variable microbiomes, not only among different species (Hubert et al., 2016b) but also among different populations of a single species (Zindel et al., 2013; Erban et al., 2017). For example, among five observed populations of mold mite [*Tyrophagus putrescentiae* (Schrank, 1781)], two populations hosted the intracellular bacterial symbiont *Cardinium* in combination with *Bacillus cereus, Staphylococcus*, and *Enterobacteriaceae*; another two populations hosted *Wolbachia* in combination with *Solitalea*-like, *Blattabacterium*-like and *Bartonella*-like bacteria; and the remaining population lacked the intracellular symbionts but was inhabited by *B. cereus, Staphylococcus*, and *Bartonella*-like bacteria (Erban et al., 2016a; Hubert et al., 2016a). These populations also differed in fitness, measured as the nutrient composition of their bodies and population growth (Erban et al., 2017).

The mite *T. putrescentiae* is a ubiquitous species living in various natural habitats, such as soil, that decomposes plant materials and vertebrate nests; *T. putrescentiae* is also very common in human-created habitats, infesting various commodities, such as wheat, oil seeds, cheese, dried ham, dried fruits, mushrooms and grain debris (Hughes, 1976), as well as dog food (Brazis et al., 2008) and fungal, plant and insect cultures in laboratories (Walter et al., 1986; Duek et al., 2001). Moreover, *Tyrophagus* mites have been reported to feed on nematodes (Walter et al., 1986; Abou El-Atta and Osman, 2016), and related species, namely, *T. similis* and *T. curvipenis*, can feed on plant leaves in greenhouses (Fain and Fauvel, 1993; Jung et al., 2010). These mites can spread dangerous fungi (e.g., those developing on grain) by carrying fungal spores on their bodies, in the digestive system, or in feces (Griffiths et al., 1959). *T. putrescentiae* is common and the second most important species (after pyroglyphid house dust mites) among house dust mites and causes allergies in people and domestic animals (Arlian et al., 1984).

The hypothesis that bacterial metacommunities influence the host food specialization in mites is linked to fungal chitin digestion and utilization by symbiotic bacteria (Smrz and Catska, 2010). Chitin-digesting enzymes produced by symbiotic bacteria have been suggested to break up structural chitin from the cell walls of filamentous fungi, which can make up a substantial part of the mites’ diet (Smrz and Catska, 1989; Erban et al., 2016b). Depending on the fungal diet, *T. putrescentiae* can have different communities of cultivable chitinolytic bacteria, *Pseudomonas, Brevundimonas*, and *Stenotrophomonas* (Smrz et al., 1991; Smrz and Trelova, 1995; Smrz and Catska, 2010). Culturing different bacterial taxa from *T. putrescentiae* fed with various diet regimes provides the first proof of bacterially assisted feeding of mites and the first evidence that bacterial communities differ among different mite diets/habitats (Smrz, 2003; Smrz and Catska, 2010). Based on these findings, one can expect different bacterial communities within a single host mite species with different diets. However, this hypothesis is not supported by manipulative experiments using diet switching (Erban et al., 2017).

Transmission of intracellular symbiotic bacteria was suggested to be vertical from mother to offspring since known (*Cardinium* and *Wolbachia*) and suspected (*Solitalea*-like, *Blattabacterium*-like) intracellular symbionts were detected inside the surface-sterilized eggs of mites (Erban et al., 2016a; Hubert et al., 2016a). Coprophagy, namely, feeding on feces, is a strategy for the acquisition of bacterial communities from feces employed by various arthropods (Nalepa et al., 2001; Engel and Moran, 2013). Although astigmatid mites do not exhibit social behavior, they naturally form aggregations on food, resulting in colonies with high densities of mite individuals (Kuwahara, 2004). In cockroaches, bacteria in feces are responsible for their host aggregation, and cockroaches are thought to acquire their gut microbiota via coprophagy (Wada-Katsumata et al., 2015). Mites’ feces contain a nitrogen waste product, guanine, which is a mite attractant (kairomone) (Levinson et al., 1991b). Mites migrate into feces-rich habitats and aggregate at these sites (Levinson et al., 1991a). In addition to guanine, astigmatid mites exhibit advanced chemical communication via various volatile secretions of the opisthosomal glands, including aggregation pheromones, which signal food availability and the presence of the same-species individuals. Massive aggregations of mites on, for example, cheese (Robertson, 1946) or dog food, are well known (Baker and Swan, 2013). Under such conditions, mite-infested habitats become covered with their feces, and feeding on the feces or feces-contaminated food may enable the acquisition of gut microbiota by mites. Mite feces are small and lightweight and may be carried by air currents along with their contents. The realization that mites’ feces serve as a vector for microorganisms is important because this strategy could be exploited for the dissemination of bacteria and fungi in soil (Griffiths et al., 1959; Brasier, 1978).

In this study, we selected two populations of *T. putrescentiae* (L and P) with different microbiomes and population growth dynamics (Erban et al., 2016a, 2017). The microbiome of L population was dominated by *Cardinium* (71% of total sequences), *Staphylococcus* (10%), *B. cereus* (10%) and *Enterobacteriaceae* with low similarity to *Xenorhabdus* (7%). In contrast, in the microbiome of P population, *Wolbachia* was the most prevalent (61%), followed by *Bartonella*-like (17%) and *Solitalea*-like species (21%) (Erban et al., 2016a, 2017). Growth medium after mite cultivation, namely, the leftover diet plus dead mite bodies and feces, was used as a source of microorganisms. Using both populations of *T. putrescentiae*, we conducted the experiments to assess the following factors: (i) microcosm experiments, in which respiration was measured in the microcosms with and without mites on mite feces-treated and control diets; (ii) the influence of feeding on a feces-enriched diet on mite fitness, measured as the amount of nutrients in the mite bodies and mite population growth; and (iii) the influence of feeding on the feces-enriched diet on the microbiome composition.

## Materials and Methods

### Experimental Mites and Spent Growth Medium for Experiments

Two populations of *T. putrescentiae* (Schrank, 1781) were used for the experiments: the L population (strain) originated from mites sampled in a grain store in Bustehrad, Bohemia, Czechia, by E. Zdarkova in 1996; the P population was obtained from specimens collected on dog food by T. Phillips in Manhattan, KS, United States, in 2014. The mites were mass-reared as previously described (Erban et al., 2016b), with the L population reared on a stored product mite wheat-derived diet (SPMd) (Erban and Hubert, 2008) and the P population on reared on ground dog kernels (DDFd) (Rybanska et al., 2016). The diet was heated at 70°C for 30 min and rehydrated (Hubert et al., 2012). Mites were removed from the cup and culture chamber surfaces and used for experiments. The spent growth medium in the chambers was used for the initial feces extract after 60 days of mite cultivation.

### Experimental Design and Samples

**FIGURE 1:**
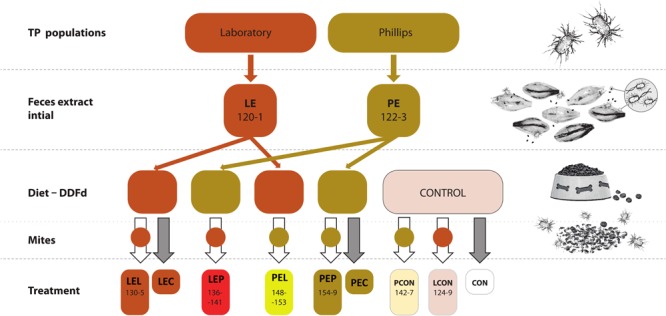
Experimental design for manipulative crosswise experiments with *Tyrophagus putrescentiae* and feces fraction. Two populations of mites (and P) were used to obtain initial extracts, which were added to the mite diets. The numbers indicate the numbers of biosamples in GenBank (Supplementary Table S1). CON, control diet without mites; LCON, control diet with mites from Laboratory population; LE, feces extract from the Laboratory population; LEC, diet treated with feces extract of mites from the Laboratory population, no mites were added; LEL, diet treated with feces extract from Laboratory population of mites with Laboratory population mites added; LEP, diet treated with feces extract from the Phillips population of mites with Laboratory population mites added; PCON, control diet with mites from the Phillips population mites; PE, feces extract from Phillips population mites; PEC, diet treated with feces extract of mites from the Phillips population mites, no mites were added; PEL, diet treated with feces extract from the laboratory population of mites with Phillips population mites added; PEP, diet treated with feces extract from the Phillips population of mites with Phillips mites added; DDFd, diet of mites from ground dog kernels.

Feces extracts were obtained from the mite-rearing chambers for L and P populations, i.e., LE and PE extracts, with six independent replicates per population (Figure 1). The chambers containing the mites and SPGM were washed with 300 mL of sterile phosphate-buffered saline (PBST – 3.2 mM Na_2_HPO_4_, 0.5 mM KH_2_PO_4_, 1.3 mM KCl, 135 mM NaCl) with 0.05% (v/v) Tween^®^ 20 detergent (Sigma-Aldrich, Saint Louis, MO, United States). The extract was transferred from the six chambers to a beaker and passed through a polyamide fiber mesh with a mesh size of 41 to 420 μm (Silk & Progress, Brnenec, Czechia) using a vacuum pump. The filtered supernatant was centrifuged (CL31R, Thermo Fisher Scientific, Waltham, MA, United States) at 845 × g for 5 min, and the supernatant was removed. The pellet was rinsed by resuspending in PBST and then centrifuged twice. The final pellet was resuspended in 10 mL of PBST. In the case of initial extract, the extracts were pooled per population for DNA extraction and diet inoculation. The extracts were applied to a powdered dog kernel; a diet lacking any extract was used in pure form as the control. Ground dog kernels (10 g) (Friskies Junior Life Plus Nutrition, Nestle Purina, Buk, Hungary) (Rybanska et al., 2016) were mixed with 4 mL of the appropriate extract; the mixtures were then vacuum dried and remoistened as previously described (Hubert et al., 2005).

Mites were introduced to the diets as follows: LCON or PCON – L or P mites fed on the control diet without any feces extracts; LEL or PEL – L or P mites fed on L mite feces extracts; LEP or PEP – L or P mites fed on P mite feces extracts. After the incubation period, the mites were removed from the rearing chambers and used for experiments. For the respiration assay, additional control treatments were prepared to include L and P-feces extract-treated diet without any mites for LEC and PEC, respectively, and control diet without mites for CON.

### The Effect of Feces Addition on Microcosm Respiration

Carbon dioxide (CO_2_) production in a microcosm consisting of mites and their diet can provide an indirect estimate of the mite feeding effect on the microbial metabolism (Hanlon and Anderson, 1979; Bengtsson and Rundgren, 1983; Siepel and Maaskamp, 1994). As shown in a preliminary experiment, mite respiration has a negligible influence on the experimental results. We conducted a respiration assay to compare CO_2_ production by diets with and without the addition of feces and mites. The following experiments were performed: CON – microcosms with diet only, no mites or feces extracts were added; microcosms with diet treated by feces extract (LE or PE) and no mites added; microcosms with mites and no feces extract treatment (LCON and PCON); and mites with feces extract-treated diet (LEL, LEP, PEL, PEP).

An infrared gas analyzer (IRGA) based on the Gascard II infrared card (Edinburgh Sensors, Livingston, SCT, United Kingdom) and a respiration apparatus (Cat No: RP1LP; Qubit systems, Kingston, ON, Canada) were used as described previously (Hubert et al., 2010). The apparatus measured CO_2_ injected from the syringe manifold. Data were collected using LabPro Interface (Cat No: C410; Qubit) and processed with LabPro 3 software (Vernier Software & Technology, Beaverton, OR, United States). The experimental design is described in Figure 1. Feces-treated and control diets were weighed to 0.01 ± 0.001 g on a Mettler AE 240 microbalance (Mettler-Toledo, Columbus, OH, United States), transferred to an Iwaki chamber to which 50 mites were added, and incubated for 7 days at 85% humidity in darkness. After incubation, the diet and mites were weighed and transferred to the syringe in the respiration manifold. The aperture of the syringe was filled with a piece of filter paper to prevent movement of the mites into the apparatus. The manifold was connected to the apparatus, and the air in the syringe was replaced with moistened synthetic gas (80% N_2_ and 20% O_2_, Cat No: GA231; Linde Gas, Prague, Czechia). The volume of the gas in every syringe was 0.6 mL. The control syringes did not contain any diets or mites. The manifolds were incubated for 2 h in an ES-500 thermostat (Trigon Plus, Cestlice, Czechia) at 25 ± 0.1°C. After the incubation, the respiration manifold was connected to the respiration apparatus, and the CO_2_ content was measured immediately via the application of 0.5 mL of gas from the syringe at 120-s intervals. Logger Pro software (Vernier, Beaverton, OR, United States) recorded the concentration of CO_2_ for 120 s. The concentration was checked 180 times per second, and 21,600 observations were collected. The total volume of CO_2_ in 0.5 mL of injected air was calculated by comparing the total sums of the IRGA–CO_2_ signals (integrals of the concentration × time curve) minus the average of the sums of the signals from the control syringes. The CO_2_ produced by the chamber over 2 h of incubation was recalculated to μL of CO_2_ per g of fresh weight for 1 h. Respiration data did not meet normality and were therefore analyzed using the Kruskal–Wallis test with a Dunn *post hoc* comparison and Bonferroni corrections.

### The Effect of Feces Addition on Mite Nutrient Contents and Population Growth

The estimation included analyses of nutrient contents in the bodies of mites, including protein, saccharide, lipid and glycogen, and a growth test. The samples included the following treatments: LEL, LEP, PEL, PEP, PCON, and LCON.

Two sets of mite culture samples were prepared. For the population growth test, 10 individuals per chamber were used, with six replicates per treatment; the experiment followed a previously described protocol (Erban et al., 2017). Although the mites underwent sexual reproduction, they were not sexed in the experiment. Separately, 100 unsexed mites were added to chambers in six replicates per treatment and cultivated for 21 days. For nutrient content analysis and DNA extraction, mites were collected from the chamber surface and plug as described above, transferred to 1.5-mL Eppendorf tubes, and weighed using a microbalance (MS Mettler-Toledo, Greifensee, Switzerland) to obtain 0.05 ± 0.01-g wet-weight samples (Erban et al., 2016a).

Nutrient content analyses were based on the Kaufmann method (Kaufmann, 2014) with modifications (Erban et al., 2017). The design included six biological and two technical replicates per treatment (2 samples per chamber). The data were processed using a previously described protocol (Erban et al., 2017). Data were analyzed with the Kruskal–Wallis test using a Dunn *post hoc* comparison and Bonferroni correction.

The growth test began by introducing 10 adult unsexed mites to treated diets; 10 replicates were performed. The mites were transferred to Iwaki flasks with 0.01 ± 0.001 g of diet and maintained under conditions described for mite cultivation (Erban et al., 2017). The experiment was terminated after 21 days, as described above, and the mites were counted under a dissecting microscope (Erban et al., 2016b). Data were analyzed with a Kruskal–Wallis test.

### The Effect of Feces on the Mite Gut Contents

Approximately 100 individuals per treatment described above were removed, added to modified Bouin–Dubosque fluid (Smrz, 1989) and used for histological observations. The mites were transferred to paraffin and sectioned using a Microm HM 200 ErgoStar Microtome (Carl Zeiss, Jena, Germany) according to a previously described protocol (Erban et al., 2016a). Slide sections were stained with Mann–Domenici stains (Exbrayat, 2013) for bacterial visualization (Supplementary Figure S1). At least 15 specimens per treatment were observed using an Axioskop compound microscope equipped with a digital camera and AxioVision software (Carl Zeiss). Gut terminology followed that used for *Acarus siro* (Sobotnik et al., 2008).

### The Effect of Feces on the Mite Microbiome

The microbiome was characterized for feces extract LE and PE, mites originated from the control cultures (LCON, PCON), and feces extract-treated diets (LEL, LEP, PEL, and PEP) (Figure 1). Samples used for mite DNA extraction originated from the same cultures as the samples used for nutrient contents experiments (see above) but were processed independently. Mites were surface cleaned, and the DNA was extracted according to a previously described protocol (Erban et al., 2016a). DNA was also extracted from 3 mL of feces extracts. DNA was isolated using a Wizard Genomic DNA Purification Kit (Cat No. A1125, Promega, Madison, WI, United States) and stored in a freezer at -28°C. We extracted DNA from every mite sample for a total of six replicates (rearing chambers) per population and two technical replicates from pooled extracts from the six rearing chambers.

DNA samples were sent to MR DNA^1^ (Shallowater, TX, United States) for sequencing of the V1–V3 portion of the 16S rRNA gene with the universal primers 27Fmod and 519Rmod (Supplementary Table S1); the Illumina MiSeq platform was used with the bTEFAP^®^ process (Chiodini et al., 2015). The forward and reverse read lengths were 300 bp. The sequences were deposited in GenBank as SUB2874221 and bioproject PRJNA394876. Lists of samples and barcodes are provided in Supplementary Table S1.

Sequencing reads were processed using MOTHUR v.1.37.1 software according to the MiSeq standard operating procedure (Schloss et al., 2009; Kozich et al., 2013) and UPARSE (Edgar, 2013). Sequences were processed as previously described (Erban et al., 2017). OTUs were identified using Ribosomal Database Project (Cole et al., 2014) training set no. 15 available from UPARSE (Edgar, 2013). Chimeras were removed via alignment to the SILVA reference database (Quast et al., 2013) and UCHIME (Edgar et al., 2011), and representative sequences for OTUs were processed using the BLASTn program on the NCBI platform (Altschul et al., 1997). The resulting identifications were based on both RDP and GenBank (Supplementary Table S3). We identified *Cardinium, Solitalea*-like and *Bartonella*-like OTUs by comparing representative sequences to previously obtained near-full-length 16S rRNA cloned sequences from TP (Erban et al., 2016a), as previously described (Erban et al., 2017). Taxonomical membership and abundance of bacterial OTUs in the samples were visually summarized in Krona (Ondov et al., 2011). Data standardization was based on the subsample created in MOTHUR using 23,081 sequences. The subsampled dataset (Supplementary Table S4) was applied for all further statistical analyses using MOTHUR and later using PAST 3.06 software (Hammer et al., 2001) and R software using the “vegan” R package (Oksanen et al., 2016).

Illumina amplicon analyses: The effects of population and treatment on OTU distribution were tested by performing two-way and one-way permutational multivariate analyses of variance (PERMANOVAs) using Jaccard and Bray–Curtis dissimilarity. The Jaccard index is based on the presence and absence of OTUs, while Bray–Curtis dissimilarity uses relative OTU abundance data in non-Euclidean space (Oksanen et al., 2016). In the one-way analysis, we compared the differences between treatments using Bonferroni-corrected *P*-values. Visualization was carried out for the dbRDA (redundancy analysis) models based on the Bray–Curtis matrix of the “vegan” package; OTU abundance data were log-transformed (log_2_) (Anderson et al., 2006). Environmental variables were selected based on the forward.sel function of the package “adespatial” (Dray et al., 2017). METASTATS (White et al., 2009) was applied to describe the effects of treatment on OTUs for each population separately in MOTHUR.

Quantitative polymerase chain reaction (qPCR) analyses were carried out using a StepOnePlus^TM^ Real-Time PCR System (Life Technologies, Grand Island, NY, United States) in 96-well plates with GoTaq qPCR Master Mix (Promega). SYBR Green (Bio-Rad Laboratories, Veenendaal, Netherlands) was used as a double-stranded DNA (dsDNA) binding dye. The protocol and primers were described previously (Erban et al., 2017); see Supplementary Table S2. We employed a design consisting of six biological replicates, each of which was analyzed with two technical replicates. The data describing the numbers of gene copies amplified by universal and specific primers were log-transformed (log10) and analyzed by PERMANOVA as described above using the Gower distance (Hammer et al., 2001). Finally, the data were separated for populations and taxa and subjected to the Kruskal–Wallis test using treatment as the comparison of treatments.

## Results

### The Effect of Feces Extracts on Microcosm Respiration

Microcosm respiration was measured on diets without feces extracts with and without mites (CON, LCON, and PCON). CO_2_ production differed significantly between the microcosms based on the control diet without mites and control diet with L mites (CON and LCON) vs. control diet with P mites (PCON) (Figure 1). Almost 60-fold higher CO_2_ production was observed in the control microcosms with P mites (PCON) than in the control diet without any mites (CON). No difference in the respiration of microcosms of L mites (LCON) and the control without mites was observed (CON). These results indicate that the activity of P mites accelerated microbial metabolism. When a feces extract was added into a microcosm, the CO_2_ production differed among the populations of mites in the microcosm. The respiration of microcosms containing L population did not differ from those without mites (i.e., LEC, LEL, and LEP). The respiration of microcosm with P mites (PEP and PEL) was three times higher than that without mites (PEC); no differences in respiration between the microcosms consisting of feces extract-treated diets without mites was found (PEC and LEC). The respiration of microcosms based on P-feces extract-treated diet and P mites (PEP) was lower than in the control experiments when P mites were fed a diet without the extract (PCON). Thus, both the feces extracts inhibited the interaction between mites and bacteria, resulting in a lower microbial respiration.

### The Effect of Feces Extracts on the Mite Nutrient Contents and Population Growth

The addition of feces extract to the diet did not influence the nutrient contents in the bodies of L and P mites (Supplementary Table S5). Although there were differences among treatments in lipid, saccharide and glycogen contents, *post hoc* comparison revealed that none of the experiments involving extract treatment (LEL, LEP, PEL, PEP) differed from the controls (LCON or PCON). There was a single exception, in which the protein content in PEP was threefold lower than that in PCON.

Population growth experiments revealed that in the control diets without any feces extracts (LCON and PCON), the growth of P mites was 1.5 higher than that in L mites. The feces extract from the L population stimulated the growth of P mites but not L mites, while P-feces extract stimulated both L and P mites. The growth of L mites was threefold higher on P extract-treated diet than on the control (LEP and LCON). The growth of P mites was fourfold greater than in the control (PEP and PCON) (Figure 1).

### The Effect of Feces Extract on the Gut Contents of Mites

**FIGURE 2:**
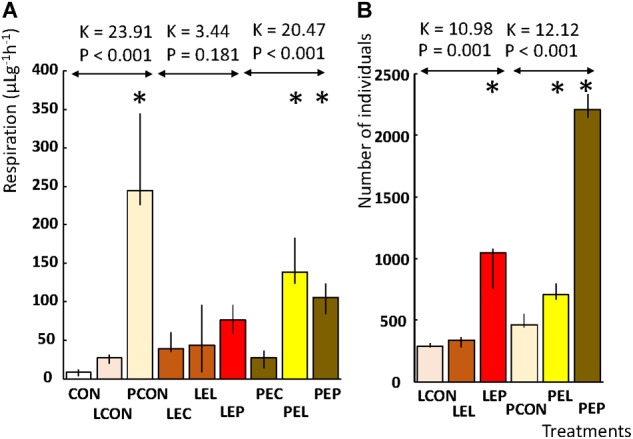
Effects of feces extract addition on the diet on the respiration and population growth of *T. putrescentiae* in a manipulative crosswise experiment. The columns are medians, and bars represent interquartile range. **(A)** Respiration is expressed as μg of CO_2_ per g of weight per hour. **(B)** Population growth was observed as the final number of individuals after 21 days from a starting population of 10 individuals; an asterisk indicates a significant difference compared to the control treatment. CON, control diet without mites; LCON, control diet with mites from the laboratory population; PCON, control diet with mites from the Phillips population; LEC, diet treated with feces extract of mites from the Laboratory population, no mites were added; PEC, diet treated with feces extract of mites from the Phillips population, no mites were added; LEL, diet treated with feces extract from the Laboratory population of mites, with laboratory mites added; LEP, diet treated with feces extract from the Phillips population of mites, with Laboratory population of mites added; PEL, diet treated with feces extract from the Laboratory population of mites, with Phillips mites added; LEP, diet treated with feces extract from the Phillips population of mites, with Phillips mites added.

Histological analyses did not detect fragments of fungal mycelium in the mite gut. There was a highly intense mesodermal cell secretion in the anterior gut, i.e., the ventriculus and the caecum (Supplementary Figure S1A). A typical food bolus (Supplementary Figure S1B) contained fragmented pieces of plant material and debris from proliferating mesodeal cells (Supplementary Figures S1B,E). Bacteria were observed attached to the surface of the food bolus (Supplementary Figure S1C) or were present in the gut lumen (Supplementary Figure S1F). There were no differences in the contents of food boli among the control mites and mites originated from feces extract-treated diets. A single exception was the food boli contained bacteria in specimens from the L population treated with P-feces extract (Figure 2). Unlike previous observations (Erban et al., 2016a), none of the specimens observed contained bacteriocytes in the fat tissues; guanine deposits in the fat tissues were observed only in specimens from the L population reared on P-feces extract-treated diet.

### Does Mite Feeding on Feces Affect Their Microbiomes?

The feces extract-treated diet had no effect on the presence/absence of OTUs in the microbiome but did influence the OTU profiles in the microbiome in both the standardized Illumina dataset and the qPCR dataset (Table 1). In the L population, feeding of mites on the diet treated by PE feces extract significantly (*P* < 0.05) altered the microbiome profile, while mite feeding on the diet treated by LE extract did not result in significant changes in the microbiome compared to that from the control (Table 1). For the P population, feeding of mites on the diets treated with both extracts (LE and PE) resulted in a significant difference in the microbial profiles from the control. In the qPCR dataset, feeding of the two populations on both feces extract-treated diets resulted in a difference in the numbers of copies of observed bacterial taxa compared to the control.

**Table 1:** Comparison (PERMANOVA) of the microbiomes of the two populations of *Tyrophagus putrescentiae* in the manipulative experiment.

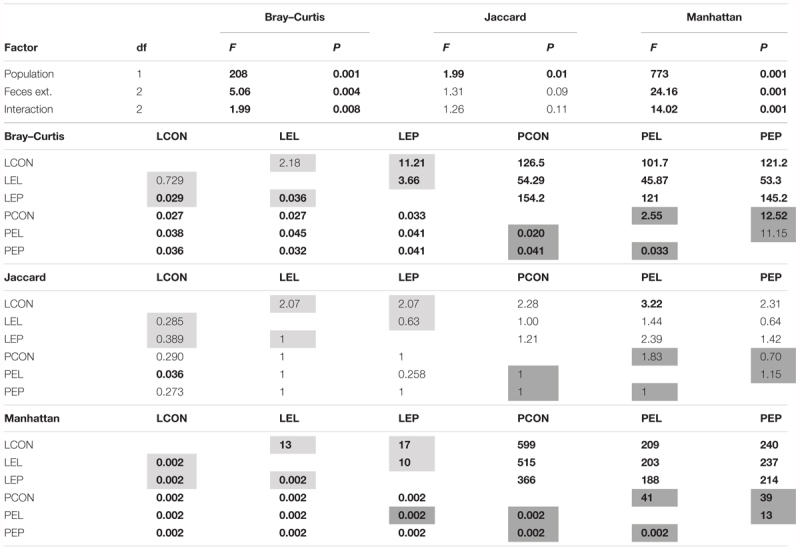

*The following two variables were tested: population (Laboratory and Phillips) and feces extracts. Pairwise comparison was performed with Bonferroni-corrected values; F-values are above the diagonal and corrected P-values below the diagonal. The bold font indicates significant differences, while the grayscale shading indicates the treatments compared. The comparison was based on the standardized Illumina amplicon data converted to log-transformed Bray–Curtis and Jaccard matrices. qPCR data used Manhattan distances*

*df, degrees of freedom; F, F-value; LCON, control diet with mites from the laboratory population; P, P-value; PCON, control diet with mites from the Phillips population; LEL, diet treated with feces extract from the Laboratory population of mites and used to rear Laboratory mites; LEP, diet treated with feces extract from the Phillips population of mites and used to rear Laboratory mites; PEL, diet treated with feces extract from the Laboratory population of mites and used to rear Phillips mites; PEP, diet treated with feces extract from the Phillips population of mites and used to rear Phillips mites*

**FIGURE 3:**
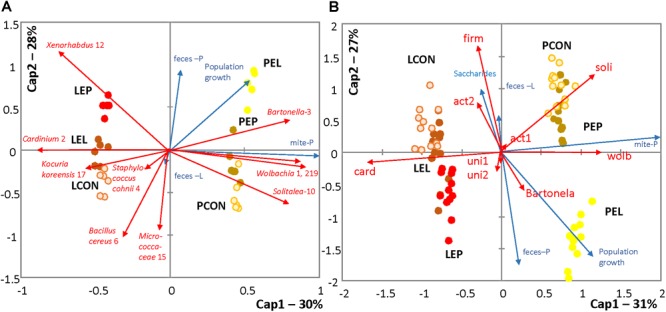
RDA triplots showing distances among profiles of the *T. putrescentiae* microbiomes under different treatments; environmental variables with a significant effect on the models are shown. **(A)** Comparison of the effects of different treatments for standardizing the Illumina dataset; the relative abundance of sequences was log-transformed (log2) and converted to a Bray–Curtis distance matrix. **(B)** Comparison of the absolute numbers of sequences obtained using species-specific and universal primers (Supplementary Table S1); the data were log-transformed (log10) and converted to a Manhattan distance matrix. LCON, control diet with mites from the laboratory population; PCON, control diet with mites from the Phillips population; LEC, diet treated with feces extract of mites from the Laboratory population, no mites were added; PEC, diet treated with feces extract of mites from the Phillips population, no mites were added; LEL, diet treated with feces extract from the Laboratory population of mites, with laboratory mites added; LEP, diet treated with feces extract from the Phillips population of mites, with Laboratory population of mites added; PEL, diet treated with feces extract from the Laboratory population of mites, with Phillips mites added; LEP, diet treated with feces extract from the Phillips population of mites, with Phillips mites added.

The log_2_-transformed OTU profiles based on Bray–Curtis distance were used to construct a dbRDA model (*F* = 31.92; *P* < 0.001; *R*^2^ = 0.87), which explained 85% of the total variance in the dataset. After forward selection of environmental variables, P population, diet treatments by both feces extracts, and population growth were significantly correlated with the model, whereas nutrient content in the bodies of mites was not. The populations were separated by the first axis and the treatments by the second axis (Figure 3A). Another dbRDA model was constructed based on qPCR data using Manhattan distances (*F* = 132.92; *P* < 0.001; *R*^2^ = 0.90), which explained 80% of the total variance. After forward variable selection, the following variables had significant effects on the model: saccharides, P population, diet treatments with both feces extracts (PE and LE) and population growth. Mite populations and treatments were separated by the first and second axes, respectively (Figure 3B).

**FIGURE 4:**
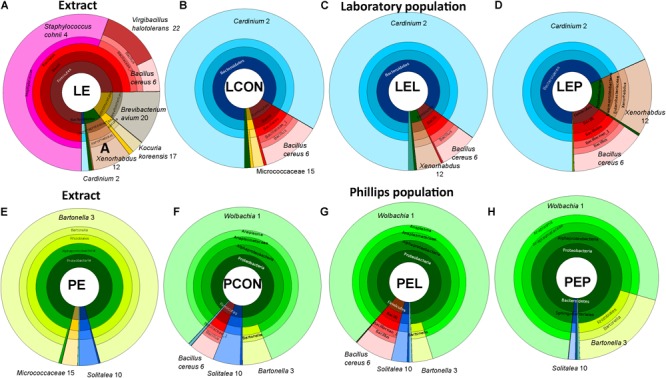
Krona visualization of microbiomes of the two populations of *T. putrescentiae* in the manipulative experiment based on feeding of mites on a diet enriched with mite feces; **(A)** bacterial composition of the feces extract form the Laboratory population (LE); **(B)** microbiome of the Laboratory population of mites fed on the control diet without any extract (LCON); **(C)** microbiome of the Laboratory population of mites fed on the Laboratory mites feces extract added to the diet (LEL); **(D)** microbiome of the Laboratory population fed on Phillips mites feces extract added to the diet (LEP); **(E)** bacterial composition of the feces extract from the Phillips population (PE); **(F)** microbiome of the Phillips population fed on the control diet without any extract added (PCON); **(G)** microbiome of the Phillips population fed on Laboratory mites feces extract added to the diet (PEL); **(H)** microbiome of the Phillips population feeding on Phillips mites feces extract added to the diet (PEP).

METASTATS analyses were used to identify which OTUs were responsible for the differences in the microbiomes (Tables 2, 3). The feces extract from the L population was composed mainly of *Staphylococcus cohnii* (OTU4), *Brevibacterium avium* (OTU20) and *Virgibacillus halotolerans* (OTU22) (Figure 4), but the relative numbers of these OTUs in both microbiomes of mites fed on extract-treated diet were not significantly different from those of the control treatments. The differences in L mites fed on L and P extract-treated diet compared to the control were as follows: an increase in *Enterobacteriaceae* with low similarity to *Xenorhabdus* (OTU12) and a decrease in *Kocuria koreensis* (OTU17) (Supplementary Figure S2). *Micrococcaceae* (OTU15) decreased in P mite feces-enriched diet (Figure 3A). The observed decrease in *Actinomycetes* of the Illumina profile was in agreement with the qPCR data (Figure 3B). The abundance of *Cardinium*, a dominant taxon in the Illumina profile (OTU2) (Figure 4), was not influenced by mite feeding on feces-treated diets, as indicated both by qPCR and Illumina amplicon datasets (Supplementary Table S6).

**Table 2:** Comparison of the effect of mite feces extract on the microbiome profile of *T. putrescentiae* Laboratory population based on METASTATS analyses of the standardized Illumina amplicon data set.

		PE		LE		LCON		LEL		LEP		LE/LEL	LCON/LEL	LCON/LEP	LELL/LEP	PE/LE	PE/LEP
OTU	OTU-identification	Mean	Stderr	Mean	Stderr	Mean	Stderr	Mean	Stderr	Mean	Stderr	*P*	*P*	*P*	*P*	*P*	*P*
OTU1	*Wolbachia*	0.49	0.18	0.28	0.01	0.32	0.05	0.29	0.04	0.37	0.03	0.882	0.753	0.409	0.095	0.288	0.672
OTU2	*Cardinium*	0.22	0.03	1.11	0.02	57.68	6.51	70.59	4.96	55.49	2.85	**0.003**	0.113	0.794	**0.015**	0.069	**0.003**
OTU6	***Bacillus cereus***	0.28	0.03	7.18	0.24	36.85	6.64	21.57	5.02	28.00	2.78	**0.042**	0.059	0.212	0.278	0.058	**0.006**
OTU4	*Staphylococcus cohnii*	0.05	0.01	58.10	1.77	1.22	0.47	0.51	0.10	0.09	0.00	**0.000**	0.130	0.028	**0.002**	**0.046**	**0.021**
OTU3	*Bartonella*-like	90.74	0.33	0.08	0.01	0.11	0.01	0.13	0.02	0.09	0.01	**0.049**	0.566	0.038	0.051	0.023	**0.001**
OTU10	*Solitalea*	4.60	0.24	0.01	0.01	0.08	0.02	0.06	0.01	0.06	0.01	**0.031**	0.183	0.122	0.864	0.092	**0.003**
OTU12	***Xenorhabdus***	0.06	0.00	6.69	0.15	0.82	0.19	**5.10**	1.21	**15.40**	1.13	0.334	**0.003**	**0.000**	**0.000**	**0.035**	**0.005**
OTU17	***Kocuria koreensis***	0.00	0.00	2.32	0.17	0.60	0.19	**0.11**	0.02	**0.15**	0.06	0.004	**0.012**	**0.030**	0.648	0.104	**0.041**
OTU15	***Micrococcaceae***	3.31	0.33	0.72	0.08	2.14	0.66	1.08	0.33	**0.18**	0.02	0.422	0.147	**0.009**	**0.012**	0.138	**0.007**
OTU22	*Virgibacillus halotolerans*	0.00	–	11.58	0.55	0.00	0.00	0.01	0.00	0.01	0.00	**0.001**	0.090	0.085	0.956	0.081	0.088
OTU219	*Wolbachia*	0.01	0.00	0.01	0.00	0.01	0.00	0.01	0.00	0.00	0.00	0.952	0.850	0.853	0.679	0.921	0.797
OTU20	***Brevibacterium avium***	–	–	10.93	1.08	0.01	0.00	**0.00**	0.00	0.00	0.00	**0.005**	**0.026**	0.057	1.000	0.115	0.578
OTU26	*Burkholderia lata*	0.09	0.01	0.23	0.02	0.05	0.01	0.08	0.02	0.05	0.01	**0.012**	0.116	0.949	0.098	0.186	**0.018**
OTU105	*Sphingopyxis macrogoltabida*	–	–	–	–	–	–	–	–	–	–	1.000	1.000	1.000	1.000	1.000	1.000
OTU103	*Chitinophagaceae*	–	–	–	–	–	–	–	–	–	–	1.000	1.000	1.000	1.000	1.000	1.000
OTU124	*Oceanobacillus neutriphilus*	–	–	–	–	–	–	–	–	–	–	1.000	1.000	1.000	1.000	1.000	1.000
OTU72	*Mesorhizobium plurifarium*	–	–	–	–	–	–	–	–	–	–	1.000	1.000	1.000	1.000	1.000	1.000
OTU41	*Anoxybacillus*	–	–	0.00	0.00	0.03	0.02	0.23	0.17	0.01	0.00	0.335	0.254	0.198	0.163	1.000	**0.043**
OTU102	*Pseudomonas plecoglossicida*	–	–	0.28	0.04	–	–	0.00	0.00	–	–	**0.007**	1.000	1.000	1.000	0.150	1.000
	**TOTAL**	**99.9**		**99.5**		**99.9**		**99.8**		**99.9**							

*OTUs were selected according to their relative numbers of the sequences. The values are in percent numbers. The bold font indicates significant differences.*

*LCON, control diet with mites from the Laboratory population; LE, feces extract from Laboratory population of mites; LEL, diet treated with feces extract from the Laboratory population of mites and used to rear Laboratory mites; LEP, diet treated with feces extract from the Phillips population of mites and used to rear Laboratory mites; P, P-values; PE, feces extract from Phillips population.*

**Table 3:** Comparison of the effect of feces extract on the microbiome profile of *T. putrescentiae* Phillips population based on METASTATS analyses of the standardized Illumina amplicon data set.

		PE		LE		LCON		LEL		LEP		LE/LEL	LCON/LEL	LCON/LEP	LELL/LEP	PE/LE	PE/LEP
OTU	OTU-identification	Mean	Stderr	Mean	Stderr	Mean	Stderr	Mean	Stderr	Mean	Stderr	*P*	*P*	*P*	*P*	*P*	*P*
OTU1	***Wolbachia***	0.49	0.18	0.28	0.01	58.13	1.57	**71.19**	4.02	**71.32**	2.01	**0.006**	**0.010**	**0.001**	0.956	0.288	**0.004**
OTU2	*Cardinium*	0.22	0.03	1.11	0.02	0.27	0.01	0.31	0.02	0.23	0.03	0.894	0.060	0.152	**0.022**	0.069	**0.002**
OTU6	*Bacillus cereus*	0.28	0.03	7.18	0.24	11.90	1.01	13.84	3.33	**0.29**	0.01	0.868	0.676	**0.000**	**0.002**	0.058	0.081
OTU4	*Staphylococcus cohnii*	0.05	0.01	58.10	1.77	0.10	0.02	0.13	0.05	0.11	0.02	**0.030**	0.579	0.606	0.725	**0.046**	**0.001**
OTU3	***Bartonella*-like**	90.74	0.33	0.08	0.01	11.47	0.59	**6.12**	0.68	**24.17**	1.65	**0.004**	**0.000**	**0.000**	**0.000**	**0.023**	**0.008**
OTU10	***Solitalea***	4.60	0.24	0.01	0.01	15.22	0.66	**5.57**	0.81	**1.86**	0.24	**0.014**	**0.000**	**0.000**	**0.001**	0.092	**0.010**
OTU12	*Xenorhabdus*	0.06	0.00	6.69	0.15	0.06	0.01	0.07	0.01	0.06	0.01	0.498	0.476	0.950	0.238	0.035	**0.000**
OTU17	*Kocuria koreensis*	0.00	0.00	2.32	0.17	0.01	0.00	0.01	0.00	0.00	0.00	0.915	0.936	0.507	0.537	0.104	**0.005**
OTU15	***Micrococcaceae***	3.31	0.33	0.72	0.08	0.93	0.09	0.75	0.13	**0.31**	0.03	**0.012**	0.280	**0.001**	**0.007**	0.138	0.916
OTU22	*Virgibacillus halotolerans*	0.00	–	11.58	0.55	0.00	0.00	0.00	0.00	0.00	0.00	0.190	0.845	0.403	0.552	0.081	**0.003**
OTU219	*Wolbachia*	0.01	0.00	0.01	0.00	1.79	0.10	1.72	0.07	1.55	0.14	**0.010**	0.691	0.177	0.236	0.921	**0.002**
OTU20	*Brevibacterium avium*	–	–	10.93	1.08	0.00	0.00	0.00	0.00	0.00	0.00	0.134	0.687	0.586	0.250	0.115	**0.006**
OTU26	*Burkholderia lata*	0.09	0.01	0.23	0.02	0.02	0.01	**0.05**	0.01	0.01	0.00	**0.007**	**0.059**	0.129	**0.016**	0.186	**0.008**
OTU105	*Sphingopyxis macrogoltabida*	–	–	–	–	–	–	–	–	–	–	1.000	1.000	1.000	1.000	1.000	1.000
OTU103	*Chitinophagaceae*	–	–	–	–	–	–	–	–	–	–	1.000	1.000	1.000	1.000	1.000	1.000
OTU124	*Oceanobacillus neutriphilus*	–	–	–	–	–	–	–	–	–	–	1.000	1.000	1.000	1.000	1.000	1.000
OTU72	*Mesorhizobium plurifarium*	–	–	–	–	–	–	–	–	–	–	1.000	1.000	1.000	1.000	1.000	1.000
OTU41	*Anoxybacillus*	–	–	0.00	0.00	0.02	0.01	0.14	0.12	0.01	0.00	0.089	0.421	0.365	0.334	1.000	0.469
OTU102	*Pseudomonas plecoglossicida*	–	–	0.28	0.04	–	–	0.00	0.00	0.00	0.00	1.000	1.000	0.455	1.000	0.150	**0.009**
	**TOTAL**	**99.86**		**99.52**		**99.92**		**99.91**		**99.92**							

*OTUs were selected according to their relative numbers of the sequences. The values are in percent numbers. The bold font indicates significant differences*.

*LCON, control diet with mites from the Laboratory population; LE, feces extract from the Laboratory population of mites; P, P-values, PE, feces extract from Phillips population; PEL, diet treated with feces extract from the Laboratory population of mites and used to rear Phillips mites; PEP, diet treated with feces extract from the Phillips population of mites and used to rear Phillips mites.*

The feces extract from P mites consisted of 91% *Bartonella* (OTU3), and the diet containing PE resulted in an increase in the relative and absolute abundance of *Bartonella* in the microbiome of the mites fed on P mites feces extract-treated diet (Figure 3A). In comparison to the microbiome of mites fed on control diet, the increase in *Bartonella* in mites reared on P-feces extract-treated diet was from 6 to 20% of the total number of sequences (Figure 4). qPCR data (Figure 4) also supported the increase in the abundance of *Bartonella* in the mites fed on P extract-treated diet. The *Wolbachia* (OTU1) profile increased in the microbiomes of mites fed on feces-enriched diets (Figures 3A,B). In contrast, the relative and absolute abundance of *Solitalea*-like bacteria decreased (Figure 4) in the microbiome of mites fed on P extract-treated diet in comparison to mites fed on the control diet.

## Discussion

### The Effect of Feces on Respiration of Microcosms With and Without Mites

Mites and their symbiotic microorganisms depend on each other, as evidenced by the increases in microbial metabolism in microcosm experiments (Hanlon and Anderson, 1979; Bengtsson and Rundgren, 1983; Siepel and Maaskamp, 1994). It is believed that the grazing of microarthropods at optimal densities accelerates microbial metabolism (Hanlon and Anderson, 1979; Bengtsson and Rundgren, 1983; Siepel and Maaskamp, 1994). Our model organism, *T. putrescentiae*, feeds on various plant materials, fungi, fungal mycelium and spores and, sometimes, on nematodes, all of which are clearly recognizable in the gut (Smrz et al., 2016). However, the respiration observed in our study was linked to bacterial metabolism because fungi were not present in the gut of mites, and fungal mycelium was not observed in the treated diet.

A new aspect of our study is the finding that different populations of *T. putrescentiae* can have different effects on microbial respiration, namely, the feeding of P mites accelerated CO_2_ production more than that of L mites in microcosm experiments. Surprisingly, the highest respiration rate was observed with the control diet (not treated with feces) after 7 days with 50 individuals of TP. The explanation is that the control diet lacked microorganisms due to pasteurization and that the mites added to this diet also inoculated a new microbial community via transfer on their bodies and feces. This microbial community has no competition with the residential bacteria. The high level of CO_2_ production in the microcosms was caused by rapid microbial growth and the rapid metabolism of available nutrients. Because the presence of mites appears to be more important than the presence of feces extracts, it is possible that the main interaction depends more on direct feeding by the mite than on the vectoring of bacteria. Although the “feces extracts” used here may not have been representative of the mite gut microbiome, as the old culture medium was washed and filtered, they still may represent a good source of feces microbiota. Nonetheless, we suggest that the bacterial taxa with the ability to colonize plant material in the diets remained, as indicated by the increase in respiration in the microcosms with feces treatment in comparison to the control without mites.

### The Effect of Feces Addition to the Diet on the Nutrient Contents in Mites and Population Growth

Mite population growth rate was suggested to be a suitable fitness indicator (Matsumoto, 1965). There were significant changes in the population growth among treatments and populations in this study. The changes in nutrients contents (lipid, glycogen, saccharide, and protein) in the mite bodies were low among the treatments. However, saccharide content was a significant factor affecting OTU distribution as evidenced by the RDA model with forward variable selection (Figure 3B).

The response of the populations of *T. putrescentiae* to feces extract addition to the diet was different. Additionally, the effect of feces extract on mite population growth differed by the origin of feces (L and P populations). The highest increase in the population growth was in P mites reared on P mite feces extract-treated diets (PEP). This increase was correlated with a positive increase in *Bartonella*-like bacteria and a decrease in *B. cereus* in the microbiome profiles. The addition of *B. cereus* to the diet decreased the population growth of *T. putrescentiae*, indicating an antagonistic effect of this bacterium on mites (Erban et al., 2016b). However, feces extracts from L mites slowly increased the growth of P mites (PEL); P mite feces extract slowly increased the growth of L mites (LEP) without any correlation with the abundance of *Bartonella*. Our explanation of these observations is that the strong bacterial growth in these experiments provided nutrients to the mites. Long-term laboratory cultivation of mites can lead to inbreeding (Wolska, 1980), which can influence mite physiology. This finding may explain the lower population growth of the L mites than of the P mites because L mites have been reared in laboratory culture for almost 30 years. Low genetic variation is indicative of inbreeding. The finding of similar ITS and CO1 haplotypes (Erban et al., 2016a) is indicative of the genetic equivalency of the cultures; thus, potential differences in response to environmental factors due to different genetic makeups can probably be ruled out.

The observed stimulatory effect of feces on the population growth may offer an explanation for the density-dependent population growth observed previously in *A. siro* (Pekar and Hubert, 2008). The rate of the increase in the *A. siro* populations was higher when starting with 100 than when starting with 50 individuals (Pekar and Hubert, 2008). Thus, the higher number of feces produced by a denser mite population enhances population growth.

### The Effect of Feces Extract Addition on the Intracellular Bacterial Symbionts of Mites

We observed changes in the profile of certain bacterial taxa among the treatments in the Illumina dataset (barcoded tag sequencing) and the qPCR dataset, which gives the absolute numbers of bacterial sequences. It is likely that certain differences in the microbiome profiles among treatments resulted from neutral processes (Burns et al., 2016) and may be interpreted as random changes in intracellular symbiont abundance (*Blattabacterium*-like, *Solitalea*-like, *Wolbachia*, and *Cardinium*).

The two populations of TP differ in the profiles of intracellular symbionts, i.e., the microbiome of the L population contains 65–85% *Cardinium* sequences, while the P population contains 78–84% of *Wolbachia* sequences. Both of these bacteria are known to affect the sexual reproduction of hosts (Werren, 1997; Zchori-Fein and Perlman, 2004). As reproductive parasites, these bacteria can occupy the same niche in different mite populations, i.e., in the reproductive organs, as in spider mites (Zhao et al., 2013), and may indicate competitive exclusion of *Wolbachia*/*Cardinium*. *Wolbachia* is able to affect the microbial species present in the fruit fly gut (*Drosophila melanogaster*) (Simhadri et al., 2017). Both intracellular symbiotic bacteria can prevent new invaders from persisting in the mite host. However, in a previous experiment, a TP population lacking intracellular symbionts did not differ from mite populations infested with intracellular symbionts (*Wolbachia* and *Cardinium*) in response to the diet perturbation (Erban et al., 2017). The present study was based on mite population-level-associated bacteria screening; the situation at the level of individuals may be different and should be studied in the future.

### The Effect of Feces Extract Addition on the *Bartonella*-Like Bacteria

Systematic (non-random) effects in the microbiome profiles were observed for *Bartonella*-like bacteria, which represented 90% of the sequences in P mite feces extracts. However, the relative and absolute numbers of the *Bartonella*-like bacteria increased in L mites after feeding on P mite feces extract-treated diet. This bacterium was present at almost 10-fold lower levels in the L microbiome than in the P microbiome. For other core taxa of the microbiome, we did not observe increases correlated with feeding on the diet treated by P mite feces extract. Thus, the change in the diet did not modulate the microbiome, as observed previously (Erban et al., 2017), even though the bacteria in feces can modulate the environment of mites.

Although we did not observe bacterial cross-contamination between the mite populations, there was a substantial horizontal transfer of *Bartonella*-like bacteria by P mites. Treatment of the diet with P mite feces extract increased the P mite fitness, and P mites increased microbial respiration. Feeding of P mites on the diet treated by P mite feces resulted in an increase in the proportion *Bartonella-*like sequences from 6 to 20% in the mite microbiome profile and accelerated mite population growth by fivefold compared to that on the untreated control.

Recent phylogenetic analyses of the 16S rRNA sequences of *Bartonella*-like bacteria (Hubert et al., 2017) have revealed that these microbes cluster outside of pathogenic *Bartonella* but together with tick-associated *Bartonella tamiae* (Billeter et al., 2008; Anderson et al., 2012) and form a sister group with *B. apis* (Kesnerova et al., 2016) together with unidentified sequences from human skin (Kong et al., 2012), ants (Bonasio et al., 2010), and stinkbugs (Matsuura et al., 2012). In ants, the bacteria have been suggested to fix atmospheric nitrogen and recycle insect waste products (Stoll et al., 2007; Anderson et al., 2012). A phylogenetic analysis of the genome of the non-pathogenic *B. apis* suggested its sister group relationship with *B. tamiae* (Segers et al., 2017). *B. apis* engages in an extracellular lifestyle and competes with other gut bacteria in nitrogen-limited plant diets (Segers et al., 2017). Our results provide evidence of an extracellular lifestyle of the *Bartonella*-like mite symbionts because these bacteria are present in relatively high numbers in the mite feces. *B. apis* possesses a conserved vitamin B12 biosynthesis pathway (Segers et al., 2017), and house dust mites require B vitamins for accelerated reproduction and growth (de Saint Georges-Gridelet, 1987). *T. putrescentiae* was observed feeding on crystalline B12 (Pankiewicz-Nowicka et al., 1986), indicating possible benefits to mite growth from the presence of B12 in *Bartonella*.

The related ant symbiotic bacterium *Ca.* Tokpelaia hoelldoblerii has the ability to recycle urea from uric acid (Neuvonen et al., 2016); however, some genes in this pathway are missing in *B. apis*, which vertically inherited the urease gene cluster to degrade urea into ammonia, which in turn is converted to glutamine and glutamate (Segers et al., 2017). Mites produce guanine as a nitrogen waste product, and guanine accumulation in fat tissues results in a pathological condition called white body symptoms. In this scenario, consumption of a nitrogen-rich diet results in accumulation of guanine crystals (Levinson et al., 1991a,b) in the fat tissue and damage to internal organs, such as the reproductive tract (Smrz and Catska, 1989; Smrz, 2003). Although we have no direct proof of the participation of *Bartonella*-like bacteria in the guanine recycling, six TP populations were compared, among which P mites showed low numbers of bodily guanine deposits (Erban et al., 2016a), corresponding to a high proportion of *Bartonella*-like bacteria in the microbiome profile. Although a comparison to the closely related symbiotic *Bartonella* indicated beneficial effects during the experiment, *Bartonella* did not exhibit proportional increases in the microbiome of L mites after treatment with PE.

Our experiments indicate that of the two predictors, the population origin and the diet, the former is the most important variable affecting the microbiome profiles. Our crosswise manipulation experiments using feces-enriched diet failed to significantly alter the microbiome composition and mite fitness. These results also highlight significant differences in the fitness of a single mite population in relation to the microbiome. *Bartonella*-like bacteria were not transferred between populations but were transferred within populations from adults to juveniles via feces. The presence of bacterial symbionts for recycling or synthesis of nitrogen compounds has not yet been proven. However, the *Bartonella*-like symbiont is a candidate for such an interaction, and further characterization of the genome of this bacterium is needed.

### The Microbiome of *T. putrescentiae* Is Resistant to the Acquisition of New Bacteria From the Feces of Mites

The recent concepts of symbiotic association between host and microbes predict that the host microbiota strongly influences the physiology, anatomy, behavior, reproduction and fitness of the host (Bordenstein and Theis, 2015; Douglas and Werren, 2016). In insects, such differences have been linked to the diet (Chandler et al., 2011; Montagna et al., 2016; Chaturvedi et al., 2017) or population origin, i.e., field or laboratory (Bansal et al., 2014). This concept also includes differences in microbiomes within a species, which can be associated with the great plasticity in the ability to consume a variety of food types in *T. putrescentiae.* In this study, however, we found that feeding on feces did not change the presence/absence of bacteria in the microbiome of *T. putrescentiae*. It is not surprising that *T. putrescentiae* populations differ in their microbiome composition and fitness among populations (Erban et al., 2017), as was also confirmed in this study. Our experiments indicate the resistance of the microbiome to change in response to feeding on a diet containing feces extracts from different populations with different microbiomes. However, the feeding on feces resulted in changes of mite fitness, namely, accelerated population growth and changes in the abundance of certain bacterial species in the mite microbiomes.

## Author’s Note

Prof. RNDr. Jaroslav Smrž, CSc., passed away suddenly on 11 August 2018 at the age of 67.

## Author Contributions

JH, TE, JS, PK, and BS carried out the scientific writing. BS and JH performed the statistical analyses and bioinformatics. MN performed the experiments and the molecular biology. JH, PK, JS, and TE executed the experimental design. JH and JS executed the microanatomy.

## Conflict of Interest Statement

The authors declare that the research was conducted in the absence of any commercial or financial relationships that could be construed as a potential conflict of interest.

## Footnotes

1 http://mrdnalab.com

## Abbreviations

CON: control diet without mites
DDFd: diet of mites from ground dog kernels
L: Laboratory population of *Tyrophagus putrescentiae*
LCON: control diet with mites from the Laboratory population
LE: feces extract from the Laboratory population
LEC: diet treated with feces extract of mites from the Laboratory population, no mites were added
LEL: diet treated with feces extract from the Laboratory population of mites with Laboratory population mites added
LEP: diet treated with feces extract from the Phillips population of mites with Laboratory population mites added
P: Phillips population of *T. putrescentiae*
PCON: control diet with mites from the Phillips population
PE: feces extract from the Phillips population
PEC: diet treated with feces extract of mites from the Phillips population, no mites were added
PEL: diet treated with feces extract from the laboratory population of mites with Phillips mites added
PEP: diet treated with feces extract from the Phillips population of mites with Phillips mites added
SPGM: spent growth medium, including the remaining contents of the culture chamber after mite cultivation, containing the diets, debris of mite bodies and mite feces
SPMd: stored product mite diet
TP: *T. putrescentiae*

## References

[B1] Abou El-Atta D. A. Osman M. A. (2016). Development and reproductive potential of *Tyrophagus putrescentiae* (Acari: Acaridae) on plant-parasitic nematodes and artificial diets. *Exp. Appl. Acarol.* 68 477–483. 10.1007/s10493-015-0002-5 26692383

[B2] Altschul S. F. Madden T. L. Schaffer A. A. Zhang J. Zhang Z. Miller W. (1997). Gapped BLAST and PSI-BLAST: a new generation of protein database search programs. *Nucleic Acids Res.* 25 3389–3402. 10.1093/nar/25.17.3389 9254694 PMC146917

[B3] Anderson K. E. Russell J. A. Moreau C. S. Kautz S. Sullam K. E. Hu Y. (2012). Highly similar microbial communities are shared among related and trophically similar ant species. *Mol. Ecol.* 21 2282–2296. 10.1111/j.1365-294x.2011.05464.x 22276952

[B4] Anderson M. J. Ellingsen K. E. McArdle B. H. (2006). Multivariate dispersion as a measure of beta diversity. *Ecol. Lett.* 9 683–693. 10.1111/j.1461-0248.2006.00926.x 16706913

[B5] Arlian L. G. Geis D. P. Vyszenski-Moher D. L. Bernstein I. L. Gallagher J. S. (1984). Antigenic and allergenic properties of the storage mite *Tyrophagus putrescentiae*. *J. Allergy Clin. Immunol.* 74 166–171. 10.1016/0091-6749(84)90281-1 6747137

[B6] Baker A. S. Swan M. C. (2013). A puzzling domestic infestation of the storage mite *Tyrophagus longior*. *J. Stored Prod. Res.* 54 64–66. 10.1016/j.jspr.2013.05.004

[B7] Bansal R. Mian M. A. R. Michel A. P. (2014). Microbiome diversity of *Aphis glycines* with extensive superinfection in native and invasive populations. *Environ. Microbiol. Rep.* 6 57–69. 10.1111/1758-2229.12108 24596263

[B8] Bengtsson G. Rundgren S. (1983). Respiration and growth of a fungus, *Mortierella isabellina*, in response to grazing by *Onychiurus armatus* (Collembola). *Soil Biol. Biochem.* 15 469–473. 10.1016/0038-0717(83)90013-5

[B9] Billeter S. A. Miller M. K. Breitschwerdt E. B. Levy M. G. (2008). Detection of two *Bartonella tamiae*-like sequences in *Amblyomma americanum* (Acari: Ixodidae) using 16S-23S intergenic spacer region-specific primers. *J. Med. Entomol.* 45 176–179. 10.1093/jmedent/45.1.176 18283962

[B10] Bonasio R. Zhang G. Ye C. Mutti N. S. Fang X. Qin N. (2010). Genomic comparison of the ants *Camponotus floridanus* and *Harpegnathos saltator*. *Science* 329 1068–1071. 10.1126/science.1192428 20798317 PMC3772619

[B11] Bordenstein S. R. Theis K. R. (2015). Host biology in light of the microbiome: ten principles of holobionts and hologenomes. *PLoS Biol.* 13:e1002226. 10.1371/journal.pbio.1002226 26284777 PMC4540581

[B12] Brasier C. M. (1978). Mites and reproduction in *Ceratocystis ulmi* and other fungi. *Trans. Br. Mycol. Soc.* 70 81–89. 10.1016/S0007-1536(78)80175-2

[B13] Brazis P. Serra M. Selles A. Dethioux F. Biourge V. Puigdemont A. (2008). Evaluation of storage mite contamination of commercial dry dog food. *Vet. Dermatol.* 19 209–214. 10.1111/j.1365-3164.2008.00676.x 18494758

[B14] Burns A. R. Stephens W. Z. Stagaman K. Wong S. Rawls J. F. Guillemin K. (2016). Contribution of neutral processes to the assembly of gut microbial communities in the zebrafish over host development. *ISME J.* 10 655–664. 10.1038/ismej.2015.142 26296066 PMC4817674

[B15] Chandler J. A. Lang J. M. Bhatnagar S. Eisen J. A. Kopp A. (2011). Bacterial communities of diverse *Drosophila* species: ecological context of a host–microbe model system. *PLoS Genet.* 7:e1002272. 10.1371/journal.pgen.1002272 21966276 PMC3178584

[B16] Chaturvedi S. Rego A. Lucas L. K. Gompert Z. (2017). Sources of variation in the gut microbial community of *Lycaeides melissa* caterpillars. *Sci. Rep.* 7:11335. 10.1038/s41598-017-11781-1 28900218 PMC5595848

[B17] Chiodini R. J. Dowd S. E. Chamberlin W. M. Galandiuk S. Davis B. Glassing A. (2015). Microbial population differentials between mucosal and submucosal intestinal tissues in advanced Crohn’s disease of the ileum. *PLoS One* 10:e0134382. 10.1371/journal.pone.0134382 26222621 PMC4519195

[B18] Cole J. R. Wang Q. Fish J. A. Chai B. McGarrell D. M. Sun Y. (2014). Ribosomal database project: data and tools for high throughput rRNA analysis. *Nucleic Acids Res.* 42 D633–D642. 10.1093/nar/gkt1244 24288368 PMC3965039

[B19] de Saint Georges-Gridelet D. (1987). Vitamin requirements of the European house dust mite, *Dermatophagoides pteronyssinus* (Acari: Pyroglyphidae), in relation to its fungal association. *J. Med. Entomol.* 24 408–411. 10.1093/jmedent/24.4.408 3625716

[B20] Douglas A. E. Werren J. H. (2016). Holes in the hologenome: why host–microbe symbioses are not holobionts. *mBio* 7:e02099. 10.1128/mBio.02099-15 27034285 PMC4817262

[B21] Dray S. Bauman D. Blanchet G. Borcard D. Clappe S. Guenard G. (2017). *Adespatial: Multivariate Multiscale Spatial Analysis*. Available at: https://cran.r-project.org/web/packages/adespatial/ [accessed March 15 2018].

[B22] Duek L. Kaufman G. Palevsky E. Berdicevsky I. (2001). Mites in fungal cultures. *Mycoses* 44 390–394. 10.1046/j.1439-0507.2001.00684.x 11766104

[B23] Edgar R. C. (2013). UPARSE: highly accurate OTU sequences from microbial amplicon reads. *Nat. Methods* 10 996–998. 10.1038/nmeth.2604 23955772

[B24] Edgar R. C. Haas B. J. Clemente J. C. Quince C. Knight R. (2011). UCHIME improves sensitivity and speed of chimera detection. *Bioinformatics* 27 2194–2200. 10.1093/bioinformatics/btr381 21700674 PMC3150044

[B25] Engel P. Moran N. A. (2013). The gut microbiota of insects – diversity in structure and function. *FEMS Microbiol. Rev.* 37 699–735. 10.1111/1574-6976.12025 23692388

[B26] Erban T. Hubert J. (2008). Digestive function of lysozyme in synanthropic acaridid mites enables utilization of bacteria as a food source. *Exp. Appl. Acarol.* 44 199–212. 10.1007/s10493-008-9138-x 18357505

[B27] Erban T. Klimov P. B. Smrz J. Phillips T. W. Nesvorna M. Kopecky J. (2016a). Populations of stored product mite *Tyrophagus putrescentiae* differ in their bacterial communities. *Front. Microbiol.* 7:1046 10.3389/fmicb.2016.01046 PMC4940368 27462300

[B28] Erban T. Rybanska D. Harant K. Hortova B. Hubert J. (2016b). Feces derived allergens of *Tyrophagus putrescentiae* reared on dried dog food and evidence of the strong nutritional interaction between the mite and *Bacillus cereus* producing protease bacillolysins and exo-chitinases. *Front. Physiol.* 7:53. 10.3389/fphys.2016.00053 26941650 PMC4764834

[B29] Erban T. Ledvinka O. Nesvorna M. Hubert J. (2017). Experimental manipulation shows a greater influence of population than dietary perturbation on the microbiome of *Tyrophagus putrescentiae*. *Appl. Environ. Microbiol.* 83:e00128-17. 10.1128/AEM.00128-17 28235879 PMC5394330

[B30] Exbrayat J.-M. (2013). *Histochemical and Cytochemical Methods of Visualization* 1st Edn. Boca Raton, FL: CRC Press 10.1201/b14967

[B31] Fain A. Fauvel G. (1993). *Tyrophagus curvipenis* n. sp. from an orchid cultivation in a greenhouse in Portugal (Acari: Acaridae). *Int. J. Acarol.* 19 95–100. 10.1080/01647959308683544

[B32] Ferrari J. Vavre F. (2011). Bacterial symbionts in insects or the story of communities affecting communities. *Philos. Trans. R. Soc. Lond. B Biol. Sci.* 366 1389–1400. 10.1098/rstb.2010.0226 21444313 PMC3081568

[B33] Griffiths D. A. Hodson A. C. Christensen C. M. (1959). Grain storage fungi associated with mites. *J. Econ. Entomol.* 52 514–518. 10.1093/jee/52.3.514

[B34] Hammer O. Harper D. A. T. Ryan P. D. (2001). PAST: paleontological statistics software package for education and data analysis. *Palaeontol. Electron.* 4 1–9. Available at: http://palaeo-electronica.org/2001_1/past/issue1_01.htm [accessed March 15 2018].

[B35] Hanlon R. D. G. Anderson J. M. (1979). The effects of collembola grazing on microbial activity in decomposing leaf litter. *Oecologia* 38 93–99. 10.1007/BF00347827 28309073

[B36] Hubert J. Doleckova-Maresova L. Hyblova J. Kudlikova I. Stejskal V. Mares M. (2005). *In vitro* and *in vivo* inhibition of alpha-amylases of stored-product mite *Acarus siro*. *Exp. Appl. Acarol.* 35 281–291. 10.1007/s10493-004-7834-8 15969461

[B37] Hubert J. Erban T. Kopecky J. Sopko B. Nesvorna M. Lichovnikova M. (2017). Comparison of microbiomes between red poultry mite populations (*Dermanyssus gallinae*): predominance of *Bartonella*-like bacteria. *Microb. Ecol.* 74 947–960. 10.1007/s00248-017-0993-z 28534089

[B38] Hubert J. Kopecky J. Nesvorna M. Perotti M. A. Erban T. (2016a). Detection and localization of *Solitalea*-like and *Cardinium* bacteria in three *Acarus siro* populations (Astigmata: Acaridae). *Exp. Appl. Acarol.* 70 309–327. 10.1007/s10493-016-0080-z 27502113

[B39] Hubert J. Kopecky J. Sagova-Mareckova M. Nesvorna M. Zurek L. Erban T. (2016b). Assessment of bacterial communities in thirteen species of laboratory-cultured domestic mites (Acari: Acaridida). *J. Econ. Entomol.* 109 1887–1896. 10.1093/jee/tow089 27122496

[B40] Hubert J. Kopecky J. Perotti M. A. Nesvorna M. Braig H. R. Sagova-Mareckova M. (2012). Detection and identification of species-specific bacteria associated with synanthropic mites. *Microb. Ecol.* 63 919–928. 10.1007/s00248-011-9969-6 22057398

[B41] Hubert J. Pekar S. Nesvorna M. Sustr V. (2010). Temperature preference and respiration of acaridid mites. *J. Econ. Entomol.* 103 2249–2257. 10.1603/ec10237 21309251

[B42] Hughes A. M. (1976). *The Mites of Stored Food and Houses: Technical Bulletin 9 of the Ministry of Agriculture, Fisheries and Food* 2nd Edn. London: Her Majesty’s Stationery Office.

[B43] Jung J.-A. Cho M.-R. Kim H.-H. Kang T.-J. Lee J.-H. Do K.-R. (2010). Damages by *Tyrophagus* similis (Acari: Acaridae) in greenhouse spinach in Korea. *Korea J. Appl. Entomol.* 49 429–432. 10.5656/ksae.2010.49.4.429

[B44] Kaufmann C. (2014). “Determination of lipid, glycogen and sugars in mosquitoes,” in *MR4 Methods in Anopheles Research* 4th Edn ed. Benedict M. (Manassas, VA: BEI Resources). Available at: https://www.beiresources.org/Publications/MethodsinAnophelesResearch.aspx [accessed March 15 2018].

[B45] Kesnerova L. Moritz R. Engel P. (2016). *Bartonella apis* sp. nov., a honey bee gut symbiont of the class *Alphaproteobacteria*. *Int. J. Syst. Evol. Microbiol.* 66 414–421. 10.1099/ijsem.0.000736 26537852

[B46] Kong H. H. Oh J. Deming C. Conlan S. Grice E. A. Beatson M. A. (2012). Temporal shifts in the skin microbiome associated with disease flares and treatment in children with atopic dermatitis. *Genome Res.* 22 850–859. 10.1101/gr.131029.111 22310478 PMC3337431

[B47] Kozich J. J. Westcott S. L. Baxter N. T. Highlander S. K. Schloss P. D. (2013). Development of a dual-index sequencing strategy and curation pipeline for analyzing amplicon sequence data on the MiSeq Illumina sequencing platform. *Appl. Environ. Microbiol.* 79 5112–5120. 10.1128/AEM.01043-13 23793624 PMC3753973

[B48] Kuwahara Y. (2004). “Chemical ecology of astigmatid mites,” in *Advances in Insect Chemical Ecology* eds Carde R. T. Millar J. G. (Cambridge: Cambridge University Press) 76–109. 10.1017/CBO9780511542664.004

[B49] Levinson H. Z. Levinson A. R. Muller K. (1991a). The adaptive function of ammonia and guanine in the biocoenotic association between ascomycetes and flour mites (*Acarus siro* L.). *Naturwissenschaften* 78 174–176. 10.1007/bf01136207

[B50] Levinson H. Z. Levinson A. R. Muller K. (1991b). Functional adaptation of two nitrogenous waste products in evoking attraction and aggregation of flour mites (*Acarus siro* L.). *Anz. Schadlingskde. Pflanzenschutz Umweltschutz* 64 55–60. 10.1007/bf01909743

[B51] Matsumoto K. (1965). Studies on environmental factors for breeding of grain mites VII. Relationship between reproduction of mites and kind of carbohydrates in the diet. *Med. Entomol. Zool.* 16 118–122. 10.7601/mez.16.118 (in Japanese with English Summary).

[B52] Matsuura Y. Kikuchi Y. Meng X. Y. Koga R. Fukatsu T. (2012). Novel clade of alphaproteobacterial endosymbionts associated with stinkbugs and other arthropods. *Appl. Environ. Microbiol.* 78 4149–4156. 10.1128/AEM.00673-12 22504806 PMC3370525

[B53] Montagna M. Mereghetti V. Gargari G. Guglielmetti S. Faoro F. Lozzia G. (2016). Evidence of a bacterial core in the stored products pest *Plodia interpunctella*: the influence of different diets. *Environ. Microbiol.* 18 4961–4973. 10.1111/1462-2920.13450 27398939

[B54] Nalepa C. A. Bignell D. E. Bandi C. (2001). Detritivory, coprophagy, and the evolution of digestive mutualisms in Dictyoptera. *Insect. Soc.* 48 194–201. 10.1007/pl00001767

[B55] Neuvonen M.-M. Tamarit D. Naslund K. Liebig J. Feldhaar H. Moran N. A. (2016). The genome of Rhizobiales bacteria in predatory ants reveals urease gene functions but no genes for nitrogen fixation. *Sci. Rep.* 6:39197. 10.1038/srep39197 27976703 PMC5156944

[B56] Oksanen J. Blanchet F. G. Kindt R. Legendre P. Minchin P. R. O’Hara R. B. (2016). *Vegan: Community Ecology Package. R package Version 2.3–5*. Available at: http://CRAN.R-project.org/package=vegan [accessed March 15 2018].

[B57] Ondov B. D. Bergman N. H. Phillippy A. M. (2011). Interactive metagenomic visualization in a web browser. *BMC Bioinformatics* 12:385. 10.1186/1471-2105-12-385 21961884 PMC3190407

[B58] Pankiewicz-Nowicka D. Boczek J. Davis R. (1986). Attraction by selected organic compounds to *Tyrophagus putrescentiae* (Acari: Acaridae). *Ann. Entomol. Soc. Am.* 79 293–299. 10.1093/aesa/79.2.293

[B59] Pekar S. Hubert J. (2008). Assessing biological control of *Acarus siro* by *Cheyletus malaccensis* under laboratory conditions: effect of temperatures and prey density. *J. Stored Prod. Res.* 44 335–340. 10.1016/j.jspr.2008.02.011

[B60] Quast C. Pruesse E. Yilmaz P. Gerken J. Schweer T. Yarza P. (2013). The SILVA ribosomal RNA gene database project: improved data processing and web-based tools. *Nucleic Acids Res.* 41 D590–D596. 10.1093/nar/gks1219 23193283 PMC3531112

[B61] Robertson P. L. (1946). Tyroglyphid mites in stored products in New Zealand. *Trans. R. Soc. N. Z.* 76 185–207.

[B62] Rybanska D. Hubert J. Markovic M. Erban T. (2016). Dry dog food integrity and mite strain influence the density-dependent growth of the stored-product mite *Tyrophagus putrescentiae* (Acari: Acaridida). *J. Econ. Entomol.* 109 454–460. 10.1093/jee/tov298 26476559

[B63] Schloss P. D. Westcott S. L. Ryabin T. Hall J. R. Hartmann M. Hollister E. B. (2009). Introducing mothur: open-source, platform-independent, community-supported software for describing and comparing microbial communities. *Appl. Environ. Microbiol.* 75 7537–7541. 10.1128/AEM.01541-09 19801464 PMC2786419

[B64] Segers F. H. I. D. Kesnerova L. Kosoy M. Engel P. (2017). Genomic changes associated with the evolutionary transition of an insect gut symbiont into a blood-borne pathogen. *ISME J.* 11 1232–1244. 10.1038/ismej.2016.201 28234349 PMC5437933

[B65] Siepel H. Maaskamp F. (1994). Mites of different feeding guilds affect decomposition of organic matter. *Soil Biol. Biochem.* 26 1389–1394. 10.1016/0038-0717(94)90222-4

[B66] Simhadri R. K. Fast E. M. Guo R. Schultz M. J. Vaisman N. Ortiz L. (2017). The gut commensal microbiome of *Drosophila melanogaster* is modified by the endosymbiont *Wolbachia*. *mSphere* 2:00287-17. 10.1128/mSphere.00287-17 28932814 PMC5597968

[B67] Smrz J. (1989). Internal anatomy of *Hypochthonius rufulus* (Acari: Oribatida). *J. Morphol.* 200 215–230. 10.1002/jmor.1052000210 29865659

[B68] Smrz J. (2003). Microanatomical and biological aspects of bacterial associations in *Tyrophagus putrescentiae* (Acari: Acaridida). *Exp. Appl. Acarol.* 31 105–113. 10.1023/B:APPA.0000005111.05959.d6 14756405

[B69] Smrz J. Catska V. (1989). The effect of the consumption of some soil fungi on the internal microanatomy of the mite *Tyrophagus putrescentiae* (Schrank) (Acari. Acaridida). *Acta Univ. Carol. Biol.* 33 81–93.

[B70] Smrz J. Catska V. (2010). Mycophagous mites and their internal associated bacteria cooperate to digest chitin in soil. *Symbiosis* 52 33–40. 10.1007/s13199-010-0099-6

[B71] Smrz J. Soukalova H. Catska V. Hubert J. (2016). Feeding patterns of *Tyrophagus putrescentiae* (Sarcoptiformes: Acaridae) indicate that mycophagy is not a single and homogeneous category of nutritional biology. *J. Insect Sci.* 16:94. 10.1093/jisesa/iew070 27638952 PMC5026478

[B72] Smrz J. Svobodova J. Catska V. (1991). Synergetic participation of *Tyrophagus putrescentiae* (Schrank) (Acari; Acaridida) and its associated bacteria on the destruction of some soil micromycetes. *J. Appl. Entomol.* 111 206–210. 10.1111/j.1439-0418.1991.tb00312.x

[B73] Smrz J. Trelova M. (1995). The association of bacteria and some soil mites (Acari: Oribatida and Acaridida). *Acta Zool. Fenn.* 196 120–123.

[B74] Sobotnik J. Alberti G. Weyda F. Hubert J. (2008). Ultrastructure of the digestive tract in *Acarus siro* (Acari: Acaridida). *J. Morphol.* 269 54–71. 10.1002/jmor.10573 17886888

[B75] Stoll S. Gadau J. Gross R. Feldhaar H. (2007). Bacterial microbiota associated with ants of the genus *Tetraponera*. *Biol. J. Linn. Soc.* 90 399–412. 10.1111/j.1095-8312.2006.00730.x

[B76] Wada-Katsumata A. Zurek L. Nalyanya G. Roelofs W. L. Zhang A. Schal C. (2015). Gut bacteria mediate aggregation in the German cockroach. *Proc. Natl. Acad. Sci. U.S.A.* 112 15678–15683. 10.1073/pnas.1504031112 26644557 PMC4697420

[B77] Walter D. E. Hudgens R. A. Freckman D. W. (1986). Consumption of nematodes by fungivorous mites, *Tyrophagus* spp. (Acarina: Astigmata: Acaridae). *Oecologia* 70 357–361. 10.1007/bf00379497 28311921

[B78] Werren J. H. (1997). Biology of *Wolbachia*. *Annu. Rev. Entomol.* 42 587–609. 10.1146/annurev.ento.42.1.587 15012323

[B79] White J. R. Nagarajan N. Pop M. (2009). Statistical methods for detecting differentially abundant features in clinical metagenomic samples. *PLoS Comput. Biol.* 5:e1000352. 10.1371/journal.pcbi.1000352 19360128 PMC2661018

[B80] Wolska K. J. (1980). Effect of inbreeding on quantitative features of copra mite *Tyrophagus putrescentiae* Schr., Acarina: Acaridae. *Genet. Pol.* 21 291–307.

[B81] Zchori-Fein E. Perlman S. J. (2004). Distribution of the bacterial symbiont *Cardinium* in arthropods. *Mol. Ecol.* 13 2009–2016. 10.1111/j.1365-294X.2004.02203.x 15189221

[B82] Zhao D.-X. Chen D.-S. Ge C. Gotoh T. Hong X.-Y. (2013). Multiple infections with *Cardinium* and two strains of *Wolbachia* in the spider mite *Tetranychus phaselus* Ehara: revealing new forces driving the spread of *Wolbachia*. *PLoS One* 8:e54964. 10.1371/journal.pone.0054964 23355904 PMC3552951

[B83] Zindel R. Ofek M. Minz D. Palevsky E. Zchori-Fein E. Aebi A. (2013). The role of the bacterial community in the nutritional ecology of the bulb mite *Rhizoglyphus robini* (Acari: Astigmata: Acaridae). *FASEB J.* 27 1488–1497. 10.1096/fj.12-216242 23307835

