# Supplementary material for: Two Populations of Mites (Tyrophagus putrescentiae) Differ in Response to Feeding on Feces-Containing Diets
Source: Front Microbiol. 2018 Oct 30;9:2590. doi: 10.3389/fmicb.2018.02590 (PMC6218854; doi:10.3389/fmicb.2018.02590)

Supplementary Material

Tittle: Two Populations of Mites (*Tyrophagus putrescentiae*) Differ in Response to Feeding on Feces-containing Diets

Authors: Jan Hubert, Marta Nesvorna, Bruno Sopko, Jaroslav Smrz, Pavel Klimov and Tomas Erban

Journal: Frontiers in Microbiology

Supplementary Tables: S1–S6

Supplementary Figures: S1-S2

Abbreviations Used

CON – control diet without mites, **LCON** –control diet with mites from Laboratory population, **PCON** – control diet with mites from Phillips population**, LEC** – diet treated with feces extract of mites from Laboratory population, without mites, **PEC** – diet treated with feces extract of mites from Phillips population, without mites, **LEL** – diet treated with feces extract from laboratory population of mites with laboratory mites, **LEP** – diet treated with feces extract from Phillips population of mites with Laboratory mites, **PEL** – diet treated with feces extract from laboratory population of mites with Phillips mites, **LEP** – diet treated with feces extract from Phillips population of mites with Phillips mites.

TABLE S1 | The list of samples used for Illumina analyses of bacterial community of *Tyrophagus putrescentiae* in different treatments. The sequences were deposited in GenBank as submission SUB2874221 and bioproject PRJNA394876. The primers and barcodes were not trimmed, the barcodes are provided.

Sample ID.	Accession	Factor	Barcode
120	SAMN07362017	LE	CGTAACCA
121	SAMN07362018	LE	CGTAAGAA
122	SAMN07362019	PE	CGTACCCA
123	SAMN07362020	PE	CGTAGATA
124	SAMN07362021	LCON	CGTAGGCT
125	SAMN07362022	LCON	CGTATTCA
126	SAMN07362023	LCON	CGTATTTC
127	SAMN07362024	LCON	CGTCAAGA
128	SAMN07362025	LCON	CGTCACAG
129	SAMN07362026	LCON	CGTCCAGG
130	SAMN07362027	LEL	CGTCGCAT
131	SAMN07362028	LEL	CGTCTGAA
132	SAMN07362029	LEL	CGTGAGAC
133	SAMN07362030	LEL	CGTGATAA
134	SAMN07362031	LEL	CGTGGGAC
135	SAMN07362032	LEL	CGTGGTCA
136	SAMN07362033	LEP	CGTTCACG
137	SAMN07362034	LEP	CGTTTACT
138	SAMN07362035	LEP	CGTTTCTA
139	SAMN07362036	LEP	CTAACACA
140	SAMN07362037	LEP	CTAACAGC
141	SAMN07362038	LEP	CTAAGAAC
142	SAMN07362039	PCON	CTAATCGC
143	SAMN07362040	PCON	CTAATGCA
144	SAMN07362041	PCON	CTACACCT
145	SAMN07362042	PCON	CTACCAAG
146	SAMN07362043	PCON	CTACGCTG
147	SAMN07362044	PCON	CTACGCTT
148	SAMN07362045	PEL	CTACTCGC
149	SAMN07362046	PEL	CTAGCTGG
150	SAMN07362047	PEL	CTAGGCAG
151	SAMN07362048	PEL	CTAGGCTA
152	SAMN07362049	PEL	CTAGTGCT
153	SAMN07362050	PEL	CTATAGTC
154	SAMN07362051	PEP	CTATCTCG
155	SAMN07362052	PEP	CTATGTCA
156	SAMN07362053	PEP	CTATTCAG
157	SAMN07362054	PEP	CTCAAACG
158	SAMN07362055	PEP	CTCAAATT
159	SAMN07362056	PEP	CTCACACT

TABLE S2 | Primers and annealing temperatures used in the study of bacterial community of *Tyrophagus putrescentiae* under various treatments.

Specificity	Name	Primer 5'–3'	Tm °C	Length bp	Reference
Universal 1	EUB338F	ACTCCTACGGGAGGCAGCAG	53	180	
	Eub518R	ATTACCGCGGCTGCTGG			Lane et al. (1991)
	Com1	CAGCAGCCGCGGTAATAC	59	270	
Universal 2	769R	ATCCTGTTTGMTMCCCVCR			Dorn-In et al. (2015)
Actinobacteria 1	Actino235F	CGCGGCCTATCAGCTTGTTG	60	283	
	Eub518R	ATTACCGCGGCTGCTGG			Stach et al. (2003)
	Acti-1154	GRDACYGCCGGGGTYAACT	59	215	
Actinobacteria 2	Acti-1339	TCWGCGATTACTAGCGAC			Pfeiffer et al. (2014)
Firmicutes	Firm934F	GGAGYATGTGGTTTAATTCGAAGCA	60	126	
	Firm1060R	AGCTGACGACAACCATGCAC			Guo et al. (2008)
	WoQf	GAAGAAGGCCTTTGGGTTGT	62	118	
Wolbachia	WoQr	AACGCTAGCCCTCTCCGTAT			Jeong et al. (2009)
Cardinium	Card_6QF	TGCAAATCTCAAAAGCATGT	54	160	
	Card_6QR	TCAAGCTCTACCAACTCCCA			Hubert et al. (2016)
Blattabacterium-like	Bla_1F	CGGCAGAAGATGGACGTG	62	190	
	Bla_1R	TACAACCCATAAGGCTGTCC			Erban et al. (2017)
	Soli_1	TGCGACACAAAGAGCTGA	56	210	
Solitalea-like	Soli_7R	TCCGCCTACACTCACCAT			Hubert et al. (2016)
	Bart_2Fq	GCCGTTGGTAGGTTTACCTA	62	200	
Bartonella-like	Bart_2R	TGTCTCCGACCCAGCCT			Erban et al. (2017)

References

Dorn-In, S., Bassitta, R., Schwaiger, K., Bauer, J., and Holzel, C. S. (2015). Specific amplification of bacterial DNA by optimized so-called universal bacterial primers in samples rich of plant DNA. *J. Microbiol. Methods* 113, 50–56. doi: 10.1016/j.mimet.2015.04.001

Erban, T., Ledvinka, O., Nesvorna, M., and Hubert, J. (2017). Experimental manipulation shows a greater influence of population than dietary perturbation on the microbiome of *Tyrophagus putrescentiae*. *Appl. Environ. Microbiol.* 83, e00128-17. doi: 10.1128/AEM.00128-17

Guo, X., Xia, X., Tang, R., Zhou, J., Zhao, H., and Wang, K. (2008). Development of a real-time PCR method for *Firmicutes* and *Bacteroidetes* in faeces and its application to quantify intestinal population of obese and lean pigs. *Lett. Appl. Microbiol.* 47, 367–373. doi: 10.1111/j.1472-765X.2008.02408.x

Hubert, J., Kopecky, J., Nesvorna, M., Perotti, M. A., and Erban, T. (2016). Detection and localization of *Solitalea*-like and *Cardinium* bacteria in three *Acarus siro* populations (*Astigmata*: *Acaridae*). *Exp. Appl. Acarol.* 70, 309–327. doi: 10.1007/s10493-016-0080-z

Jeong, G., and Stouthamer, R. (2009). Quantification of *Wolbachia* copy number in *Trichogramma* eggs (*Hymenoptera*: *Trichogrammatidae*): lysozyme treatment significantly improves total gene yield from the Gram-negative bacterium. *Entomol. Res.* 39, 66–69. doi: 10.1111/j.1748-5967.2009.00200.x

Lane, D. J. (1991). "16s/23s rRNA sequencing," in *Nucleic Acid Techniques in Bacterial Systematics*, eds. E. Stackebrandt and M. Goodfellow (Chichester & New York, NY: John Wiley and Sons), 115–175.

Pfeiffer, S., Pastar, M., Mitter, B., Lippert, K., Hackl, E., Lojan, P., et al. (2014). Improved group-specific primers based on the full SILVA 16S rRNA gene reference database. *Environ. Microbiol.* 16, 2389–2407. doi: 10.1111/1462-2920.12350

Stach, J. E. M., Maldonado, L. A., Ward, A. C., Goodfellow, M., and Bull, A. T. (2003). New primers for the class *Actinobacteria*: application to marine and terrestrial environments. *Environ. Microbiol.* 5, 828–841. doi: 10.1046/j.1462-2920.2003.00483.x

TABLE S3 | Microbiome of *T. putrescentiae*, the description of bacterial taxa identified in microbiome based on operational taxonomic units (OTU) on 3% dissimilarity level and OTUs identification based on Ribosomal database project (RDP) and comparison of representative sequences with the sequences in GenBank by Blast. Color marks the levels of confidence thresholds (c.t.), similarity (s.) and total number of sequences (N(OTU)) in standardized dataset.

ID.	phylum	c.t.	class	c.t.	order	c.t.	family	c.t.	genus	c.t.	Taxon	s (%)	I.D.	N(OTU)
OTU1	*Proteobacteria*	1	*Alphaproteobacteria*	0.7	*Rickettsiales*	0.29	*Anaplasmataceae*	0.13	*Anaplasma*	0.02	*Wolbachia*	99	KX022256	272,197
OTU2	*Bacteroidetes*	0.81	*Sphingobacteriia*	0.02	*Sphingobacteriales*	0.01	*Cyclobacteriaceae*	0	*Cyclobacterium*	0	*Cardinium*	99	KX022134	264,615
OTU3	*Proteobacteria*	1	*Alphaproteobacteria*	0.96	*Rhizobiales*	0.69	*Bartonellaceae*	0.43	*Bartonella*	0.18	*Bartonella*-like	99	KX022318	132,715
OTU4	*Firmicutes*	1	*Bacilli*	1	*Bacillales*	0.99	*Staphylococcaceae*	0.99	*Staphylococcus*	0.99	*Staphylococcus cohnii*	99	NR_037046	158,859
OTU6	*Firmicutes*	1	*Bacilli*	1	*Bacillales*	0.99	*Bacillaceae_1*	0.98	*Bacillus*	0.98	*Bacillus cereus*	99	NR_074540	231,635
OTU9	*Bacteroidetes*	0.56	*Flavobacteriia*	0.02	*Flavobacteriales*	0.01	*Flavobacteriaceae*	0	*Tenacibaculum*	0	*Blattabacterium*-like	99	KX022159	341
OTU10	*Bacteroidetes*	0.93	*Sphingobacteriia*	0.3	*Sphingobacteriales*	0.15	*Sphingobacteriaceae*	0.07	*Mucilaginibacter*	0.01	*Solitale*	83	NR_109525	45,560
OTU11	*Actinobacteria*	1	*Actinobacteria*	1	*Actinomycetales*	0.98	*Brevibacteriaceae*	0.92	*Brevibacterium*	0.88	*Brevibacterium siliguriense*	98	NR_115086	218
OTU12	*Proteobacteria*	1	*Gammaproteobacteria*	0.97	*Enterobacteriales*	0.82	*Enterobacteriaceae*	0.62	*Xenorhabdus*	0.08	*Xenorhabdus*	92	NR_042325	42,497
OTU15	*Actinobacteria*	1	*Actinobacteria*	0.97	*Actinomycetales*	0.87	*Micrococcaceae*	0.39	*Arthrobacter*	0.09	*Acaricomes phytoseiuli*	90	NR_042334	13,412
OTU17	*Actinobacteria*	1	*Actinobacteria*	1	*Actinomycetales*	0.99	*Micrococcaceae*	0.99	*Kocuria*	0.98	*Kocuria koreensis*	99	NR_116745	18,277
OTU20	*Actinobacteria*	1	*Actinobacteria*	1	*Actinomycetales*	0.98	*Brevibacteriaceae*	0.91	*Brevibacterium*	0.81	*Brevibacterium avium*	97	NR_026485	5,242
OTU22	*Firmicutes*	1	*Bacilli*	0.97	*Bacillales*	0.87	*Bacillaceae_1*	0.39	*Bacillus*	0.13	*Virgibacillus halotolerans*	99	NR_108860	8,975
OTU26	*Proteobacteria*	1	*Betaproteobacteria*	1	*Burkholderiales*	0.99	*Burkholderiaceae*	0.99	*Burkholderia*	0.97	*Burkholderia lata*	99	NR_102890	1,875
OTU34	*Actinobacteria*	1	*Actinobacteria*	0.95	*Solirubrobacterales*	0.82	*Solirubrobacteraceae*	0.5	*Solirubrobacter*	0.28	*Solirubrobacter ginsenosidimutans*	92	NR_108192	310
OTU38	*Firmicutes*	1	*Bacilli*	1	*Lactobacillales*	1	*Lactobacillaceae*	1	*Lactobacillus*	1	*Lactobacillus amylovorus*	99	NR_043287	211
OTU41	*Firmicutes*	1	*Bacilli*	1	*Bacillales*	0.99	*Bacillaceae_1*	0.98	*Anoxybacillus*	0.97	*Anoxybacillus flavithermus*	91	NR_026516	633
OTU43	*Proteobacteria*	1	*Alphaproteobacteria*	1	*Rhizobiales*	0.99	*Bradyrhizobiaceae*	0.91	*Afipia*	0.78	*Afipia birgiae*	99	NR_025117	496
OTU72	*Proteobacteria*	1	*Alphaproteobacteria*	1	*Rhizobiales*	0.94	*Phyllobacteriaceae*	0.74	*Mesorhizobium*	0.58	*Mesorhizobium plurifarium*	98	NR_114124	709
OTU94	*Firmicutes*	1	*Bacilli*	0.96	*Bacillales*	0.84	*Bacillaceae_2*	0.35	*Filobacillus*	0.07	*Virgibacillus campisalis*	91	NR_108840	217
OTU102	*Proteobacteria*	1	*Gammaproteobacteria*	1	*Pseudomonadales*	0.99	*Pseudomonadaceae*	0.99	*Pseudomonas*	0.98	*Pseudomonas plecoglossicida*	99	NR_114226	604
OTU103	*Bacteroidetes*	1	*Sphingobacteriia*	0.99	*Sphingobacteriales*	0.97	*Chitinophagaceae*	0.95	*Sediminibacterium*	0.04	*Hydrobacter penzbergensis*	95	NR_134746	917
OTU105	*Proteobacteria*	1	*Alphaproteobacteria*	1	*Sphingomonadales*	0.97	*Sphingomonadaceae*	0.94	*Sphingopyxis*	0.85	*Sphingopyxis macrogoltabida*	99	NR_113720	963
OTU123	*Proteobacteria*	1	*Alphaproteobacteria*	0.94	*Rhizobiales*	0.44	*Methylobacteriaceae*	0.19	*Methylobacterium*	0.05	*Nordella oligomobilis*	99	NR_114615	222
OTU124	*Firmicutes*	1	*Bacilli*	1	*Bacillales*	0.99	*Bacillaceae_2*	0.98	*Oceanobacillus*	0.98	*Oceanobacillus neutriphilus*	99	NR_116424	852
OTU154	*Firmicutes*	1	*Bacilli*	0.99	*Bacillales*	0.95	*Bacillaceae_1*	0.74	*Bacillus*	0.58	*Bacillus galactosidilyticus*	96	NR_025580	457
OTU165	*Proteobacteria*	1	*Alphaproteobacteria*	1	*Caulobacterales*	0.97	*Caulobacteraceae*	0.94	*Caulobacter*	0.71	*Sphingomonas leidyi*	99	NR_025324	166
OTU186	*Proteobacteria*	1	*Gammaproteobacteria*	1	*Pseudomonadales*	0.99	*Pseudomonadaceae*	0.99	*Pseudomonas*	0.98	*Pseudomonas migulae*	99	NR_114223	244
OTU207	*Firmicutes*	1	*Bacilli*	1	*Bacillales*	0.99	*Bacillaceae_2*	0.98	*Oceanobacillus*	0.98	*Oceanobacillus oncorhynchi*	99	NR_042257	166
OTU219	*Proteobacteria*	1	*Alphaproteobacteria*	0.64	*Rickettsiales*	0.26	*Anaplasmataceae*	0.12	*Anaplasma*	0.01	*Anaplasma phagocytophilum*	90	NR_044762	6,882
OTU225	*Firmicutes*	1	*Bacilli*	0.95	*Bacillales*	0.82	*Bacillaceae_2*	0.35	*Virgibacillus*	0.06	*Virgibacillus campisalis*	91	NR_108840	178
OTU239	*Actinobacteria*	1	*Actinobacteria*	1	*Actinomycetales*	0.99	*Mycobacteriaceae*	0.97	*Mycobacterium*	0.98	*Mycobacterium brisbanense*	99	NR_029037	368
OTU313	*Proteobacteria*	1	*Gammaproteobacteria*	1	*Pseudomonadales*	0.98	*Moraxellaceae*	0.99	*Acinetobacter*	0.98	*Acinetobacter bereziniae*	99	NR_117625	207
OTU323	*Proteobacteria*	1	*Alphaproteobacteria*	1	*Rhizobiales*	0.97	*Brucellaceae*	0.85	*Ochrobactrum*	0.64	*Ochrobactrum anthropi*	98	NR_074243	140
OTU393	*Proteobacteria*	1	*Betaproteobacteria*	1	*Burkholderiales*	0.95	*Oxalobacteraceae*	0.92	*Naxibacter*	0.58	*Massilia alkalitolerans*	97	NR_043094	306

TABLE S4 | Microbiome of *T. putrescentiae* obtained from Illumina amplicon sequencing the list of samples and the profile of OTUs. The data were standardized and the numbers sequences in standardized data set are showed. Color marks the levels of abundance values.

	Treatment	LCON	LCON	LCON	LCON	LCON	LCON	LEL	LEL	LEL	LEL	LEL	LEL	LEP	LEP	LEP	LEP	LEP	LEP
OTUs97	Sample ID	124	125	126	127	128	129	130	131	132	133	134	135	136	137	138	139	140	141
OTU1	*Wolbachia*	118	87	81	36	47	83	64	39	87	54	88	86	75	108	72	73	120	86
OTU2	*Cardinium*	16,722	11,240	10,814	9,508	14,968	19,121	16,974	11,235	19,378	17,082	18,740	17,399	11,999	16,182	12,561	12,202	14,156	12,142
OTU3	*Bartonella*-like	31	22	23	23	32	31	34	22	21	50	28	29	22	21	17	22	23	26
OTU4	*Staphylococcus cohnii*	18	224	760	414	288	33	107	90	37	114	179	205	22	20	20	27	24	20
OTU6	*Bacillus cereus*	5,190	11,526	11,219	13,352	7,708	3,624	5,128	10,338	2,368	5,835	2,403	4,729	7,861	3,868	8,371	7,229	5,875	6,779
OTU9	*Blattabacterium*-like	8	10	11	7	7	11	14	9	16	6	14	10	10	10	14	7	14	11
OTU10	*Solitalea*	14	18	11	32	30	16	15	19	4	10	16	23	23	7	12	15	13	12
OTU11	*Brevibacterium siliguriense*	3	8	8	4	3	3	3	1	8	3	4	6	9	2	6	4	6	4
OTU12	*Xenorhabdus*	348	99	127	80	213	305	1,288	1,828	776	206	2,143	1,045	3,687	3,511	2,635	4,156	3,441	4,559
OTU15	*Micrococcaceae*	1,262	395	402	144	366	487	130	144	599	384	113	178	37	42	53	24	54	42
OTU17	*Kocuria koreensis*	64	152	331	180	110	22	16	34	40	33	13	20	38	1	15	4	65	84
OTU20	*Brevibacterium avium*	0	2	1	2	2	5	0	1	0	0	0	1	0	0	0	2	1	0
OTU22	*Virgibacillus halotolerans*	1	1	1	1	2	1	1	1	5	1	2	5	1	2	6	3	1	2
OTU26	*Burkholderia lata*	13	5	7	6	14	22	13	18	13	8	34	24	12	14	10	21	5	5
OTU34	*Solirubrobacter*	0	0	0	0	0	0	0	0	0	0	0	0	0	0	0	0	0	0
OTU38	*Lactobacillus amylovorus*	1	0	0	0	1	0	0	1	0	0	0	0	1	0	0	0	0	0
OTU41	*Anoxybacillus*	7	1	0	2	4	29	4	13	259	7	13	28	1	4	1	1	0	1
OTU43	*Afipia birgiae*	0	0	0	0	0	0	0	0	0	0	0	0	0	0	0	0	0	0
OTU72	*Mesorhizobium plurifarium*	0	0	0	0	0	0	0	0	0	0	0	0	0	0	0	0	0	0
OTU94	*Virgibacillus*	0	0	0	0	0	0	0	0	0	0	0	0	0	0	0	0	0	0
OTU102	*Pseudomonas plecoglossicida*	0	0	0	0	0	0	0	1	0	0	0	0	0	0	0	0	0	0
OTU103	*Chitinophagaceae*	0	0	0	0	0	0	0	0	0	0	0	0	0	0	0	0	0	0
OTU105	*Sphingopyxis macrogoltabida*	0	0	0	0	0	0	0	0	0	0	0	0	0	0	0	0	0	0
OTU123	*Nordella oligomobilis*	0	0	0	0	0	0	0	0	0	0	0	0	0	0	0	0	0	0
OTU124	*Oceanobacillus neutriphilus*	0	0	0	0	0	0	0	0	0	0	0	0	0	0	0	0	0	0
OTU154	*Bacillus*	0	0	0	0	0	0	0	0	0	0	0	0	0	0	0	0	0	0
OTU165	*Sphingomonas leidyi*	0	0	0	0	0	0	0	0	0	0	0	0	0	0	0	0	0	0
OTU186	*Pseudomonas migulae*	0	0	1	0	0	2	1	2	1	0	1	2	1	0	0	0	0	1
OTU207	*Oceanobacillus oncorhynchi*	0	0	0	0	0	0	0	0	0	0	0	0	0	0	0	0	0	0
OTU219	*Wolbachia*	0	4	0	3	1	0	2	1	1	1	3	1	0	2	0	3	0	2
OTU225	*Bacillaceae_2*	0	0	0	0	0	0	0	0	0	0	0	0	0	0	0	0	0	0
OTU239	*Mycobacterium brisbanense*	0	0	0	0	0	0	0	0	0	0	0	0	0	0	0	0	0	0
OTU313	*Acinetobacter bereziniae*	0	0	0	0	0	1	0	0	0	0	0	0	0	0	0	0	0	0
OTU323	*Ochrobactrum anthropi*	0	0	0	0	0	0	0	0	0	0	0	0	0	0	0	0	0	0
OTU393	*Massilia alkalitolerans*	0	0	0	0	0	0	0	0	0	0	0	0	0	0	0	0	0	0

TABLE S4 continuation | Microbiome of *T. putrescentiae* obtained from Illumina amplicon sequencing the list of samples and the profile of OTUs. The data were standardized and the numbers sequences in standardized data set are showed. Color marks the levels of abundance values.

	Treatment	PCON	PCON	PCON	PCON	PCON	PCON	PEL	PEL	PEL	PEL	PEl	PEL	PEP	PEP	PEP	PEP	PEP
OTUs97	Sample ID	142	143	144	145	146	147	148	149	150	151	152	153	154	156	157	158	159
OTU1	*Wolbachia*	13,180	13,365	14,859	14,370	14,640	12,595	16,833	13,521	15,179	18,847	17,386	19,903	15,167	17,650	17,424	17,779	16,856
OTU2	*Cardinium*	71	71	58	59	60	68	69	73	72	91	68	66	52	75	34	52	57
OTU3	*Bartonella-like*	2,597	2,976	2,374	2,362	3,222	2,842	1,866	1,305	1,934	1,169	1,548	924	7,202	5,179	5,373	5,063	5,948
OTU4	*Staphylococcus cohnii*	26	35	12	14	32	21	83	18	44	13	16	14	30	19	35	31	16
OTU6	*Bacillus cereus*	3,048	3,023	2,647	2,837	1,827	3,618	2,970	6,839	4,116	1,785	2,118	1,942	79	69	61	64	68
OTU9	*Blattabacterium-like*	6	4	4	8	7	3	3	9	5	9	8	7	9	16	5	18	9
OTU10	*Solitalea*	4,103	3,683	3,155	3,570	3,242	3,987	1,296	1,381	1,675	1,355	1,790	452	671	379	408	393	359
OTU11	*Brevibacterium siliguriense*	3	2	1	7	3	2	6	7	1	3	5	6	2	8	1	1	3
OTU12	*Xenorhabdus*	5	13	11	22	17	14	11	20	18	23	13	13	19	13	11	12	13
OTU15	*Micrococcaceae*	269	178	284	156	220	221	182	209	293	99	205	90	83	94	76	53	62
OTU17	*Kocuria koreensis*	3	1	2	0	1	1	1	3	1	2	0	1	0	3	0	0	1
OTU20	*Brevibacterium avium*	1	1	1	0	1	0	1	0	1	0	0	0	1	1	0	3	0
OTU22	*Virgibacillus halotolerans*	0	1	0	4	1	1	0	1	0	0	3	2	1	1	0	1	0
OTU26	*Burkholderia lata*	3	7	2	7	10	2	10	5	8	25	10	10	4	4	2	1	3
OTU34	*Solirubrobacter*	0	0	0	0	0	0	0	0	0	0	0	0	0	0	0	0	0
OTU38	*Lactobacillus amylovorus*	0	1	0	1	0	0	1	0	1	0	0	0	1	1	0	1	0
OTU41	*Anoxybacillus*	3	0	4	8	1	10	0	8	1	2	180	2	1	1	1	2	7
OTU43	*Afipia birgiae*	0	0	0	0	0	0	0	0	0	0	0	0	0	0	0	0	0
OTU72	*Mesorhizobium plurifarium*	0	0	0	0	0	0	0	0	0	0	0	0	0	0	0	0	0
OTU94	*Virgibacillus*	0	0	0	0	0	0	0	0	0	0	0	0	0	0	0	0	0
OTU102	*Pseudomonas plecoglossicida*	0	0	0	0	0	0	0	0	0	1	0	0	0	1	0	0	0
OTU103	*Chitinophagaceae*	0	0	0	0	0	0	0	0	0	0	0	0	0	0	0	0	0
OTU105	*Sphingopyxis macrogoltabida*	0	0	0	0	0	0	0	0	0	0	0	0	0	0	0	0	0
OTU123	*Nordella oligomobilis*	0	0	0	0	0	0	0	0	0	0	0	0	0	0	0	0	0
OTU124	*Oceanobacillus neutriphilus*	0	0	0	0	0	0	0	0	0	0	0	0	0	0	0	0	0
OTU154	*Bacillus*	0	0	0	0	0	0	0	0	0	0	0	0	0	0	0	0	0
OTU165	*Sphingomonas leidyi*	0	0	0	0	0	0	0	0	0	0	0	0	0	0	0	0	0
OTU186	*Pseudomonas migulae*	0	0	0	0	0	0	1	1	1	0	2	1	1	0	1	0	0
OTU207	*Oceanobacillus oncorhynchi*	0	0	0	0	0	0	0	0	0	0	0	0	0	0	0	0	0
OTU219	*Wolbachia*	475	432	359	365	509	411	464	395	446	369	420	367	474	282	366	323	394
OTU225	*Bacillaceae_2*	0	0	0	0	0	0	0	0	0	0	0	0	0	0	0	0	0
OTU239	*Mycobacterium brisbanense*	0	0	0	0	0	0	0	0	0	0	0	0	0	0	0	0	0
OTU313	*Acinetobacter bereziniae*	0	0	0	0	0	0	0	0	0	0	0	0	1	0	0	0	0
OTU323	*Ochrobactrum anthropi*	0	0	0	0	0	0	0	0	0	0	0	0	0	0	0	0	0
OTU393	*Massilia alkalitolerans*	0	0	0	0	0	0	0	0	0	0	0	0	0	0	0	0	0

TABLE S5 | The comparison of nutrient status of *T. putrescetiae* in manipulative crosswise experiment. The data (mg/g of fresh weight) are presented as medians and interquartile ranges in brackets. The statistical analyses included Kruskal–Wallis test and Dunn potshot comparison after Bonferroni correction.

**Nutrient**	**Treatment**	**Median**	**Intq. range**	**DF**	**K**	**P**
Lipids	LCON	67a	(59–72)	5	11.07	0.0003
	LEL	75b	(65–72)			
	LEP	66b	(62–75)			
	PCON	68b	(60–80)			
	PEL	77b	(68–84)			
	PEP	30b	(25–41)			
Saccharides	LCON	4.72a	(3.36–5.69)	5	11.07	0.0020
	LEL	5.52ab	(3.96–6.21)			
	LEP	6.05ab	(5.15–6.94)			
	PCON	5.73b	(4.28–7.07)			
	PEL	5.93b	(4.28–8.20)			
	PEP	1.97b	(1.45–2.55)			
Glycogen	LCON	47ab	(41–51)	5	33.559	< 0.001
	LEL	41a	(36–49)			
	LEP	74bc	(58–84)			
	PCON	75c	(65–86)			
	PEL	85c	(69–98)			
	PEP	58abc	(46–60)			
Proteins	LCON	198ab	(141–291)	5	13.859	0.017
	LEL	265ab	(224–296)			
	LEP	236ab	(219–267)			
	PCON	328b	(287–356)			
	PEL	291b	(283–454)			
	PEP	113a	(84–152)			

TABLE S6 | Description of the microbiome of *Tyrophagus putrescentiae* under experimental addition of feces extract to the diet using qPCR and taxa-specific primers (see Table S2). The data describing the numbers of copies per mite were Log-transformed and compared by applying the Kruskal–Wallis test for each population separately and are presented for the Laboratory and Phillips population below. The data are displayed as medians and interquartile ranges. The letters indicated a significant difference in post hot comparison.

Tretment	LCON		LEL		LEP		K	P
Taxon	Med.	Int.ran.	Med.	Int.ran.	Med.	Int.ran.		
Univ. 1	5.00ab	(4.73–5.13)	4.81a	(4.77–4.88)	5.01b	(4.92–5.06)	9.15	0.01
Univ. 2	5.81a	(5.56–5.84)	5.67a	(5.62–5.77)	5.88b	(5.82–5.94)	15.75	0.001
Act. 1.	4.15b	(3.59–4.34)	3.50a	(3.42–3.62)	3.73ab	(3.53–3.91)	8.52	0.014
Act. 2.	7.56b	(7.28–7.76)	6.84a	(6.67–6.94)	6.75a	(6.11–7.08)	17.17	0.001
Firm	3.81b	(3.52-4.05)	3.50a	(3.43–3.61)	3.76b	(3.70–3.81)	13.55	0.001
Solitalea	0.55a	(0.19–0.62)	0.50a	(0.40–0.56)	0.82b	(0.74–0.89)	17.85	0.001
Cardinium	3.82	(3.62–3.92)	3.6	(3.56–3.63)	3.61	(3.57–3.65)	5.43	0.067
Wolbachia	1.37a	(1.08–1.57)	1.75b	(1.63–2.01)	2.23b	(2.20–2.40)	22.38	<0.001
Bartonella	2.95a	(2.87–2.98)	3.00a	(3.00–3.08)	3.14b	(3.07–3.16)	20.86	<0.001
Tretment	PCON		PEL		PEP			
Univ. 1	4.94b	(4.85–5.10)	4.71a	(4.62–4.81)	4.96b	(4.95–5.03)	18.26	0.001
Univ. 2	5.72b	(5.68–5.91)	5.50a	(5.46–5.60)	5.83b	(5.84–5.86)	18.99	<0.001
Act. 1.	3.56a	(3.52–3.73)	3.90b	(3.87–3.93)	3.75ab	(3.68–3.93)	10.36	0.006
Act. 2.	6.71b	(6.52–6.78)	6.42a	(6.13–6.62)	6.39a	(6.29–6.48)	11.75	0.003
Firm	3.59b	(3.53–3.67)	3.45b	(3.35–3.61)	2.40a	(2.36–2.45)	23.89	<0.001
Solitalea	3.31b	(3.24–3.41)	2.88a	(2.79–2.91)	2.74a	(2.65–2.80)	25.52	<0.001
Cardinium	0.65b	(0.31–0.75)	0.58b	(0.02–0.87)	0.02a	(0.01–0.02)	10.95	0.004
Wolbachia	4.10b	(4.06–4.25)	3.95a	(3.81–4.03)	4.18b	(4.16–4.19)	17.82	0.001
Bartonella	3.48a	(3.44–3.64)	3.28a	(3.19–3.40)	3.85b	(3.83–3.89)	28.24	<0.001

FIGURE S1 | Mann–Dominici stained histological sections of *Tyrophagus putrescentiae* individuals after different treatments. **A** – Laboratory population, control treatment (LCON): total view of the digestive tract; arrows indicate proliferating caecal and ventricular cells. **B** – Phillips population, Phillips feces extract treatment: detailed view of a food bolus containing anamorphous food contents; arrows indicate proliferating apical parts of the ventricular cells entering the gut lumen. **C** – Laboratory population, control treatment (LCON): detailed view of a food bolus containing anamorphous food particles with bacteria attached to the surface of the bolus (arrow). **D** – Laboratory population, Phillips feces extract treatment (LEP): detailed view of a food bolus formed by bacteria only (arrows). **E** – Phillips population, Laboratory population feces extract treatment (PEL): total view of the postcolon and anal atrium; the food bolus contains anamorphous food particles and debris from the proliferating mesodeal cells, as indicated by arrows. **F** – Phillips population, control treatment (PCON): detailed view of a food bolus in the postcolon; the food bolus contains anamorphous food particles, bacteria (arrows) are attached to the food bolus surface and are present in the gut lumen.

Legend: ac – anal atrium with cuticle, ca – caecum, c – cuticle, co – colon, fb – food bolus, ph – pharynx, ic – inter colon, o – esophagus, sg – synganglion, v – ventriculus, **Scale bars:** 25 µm.

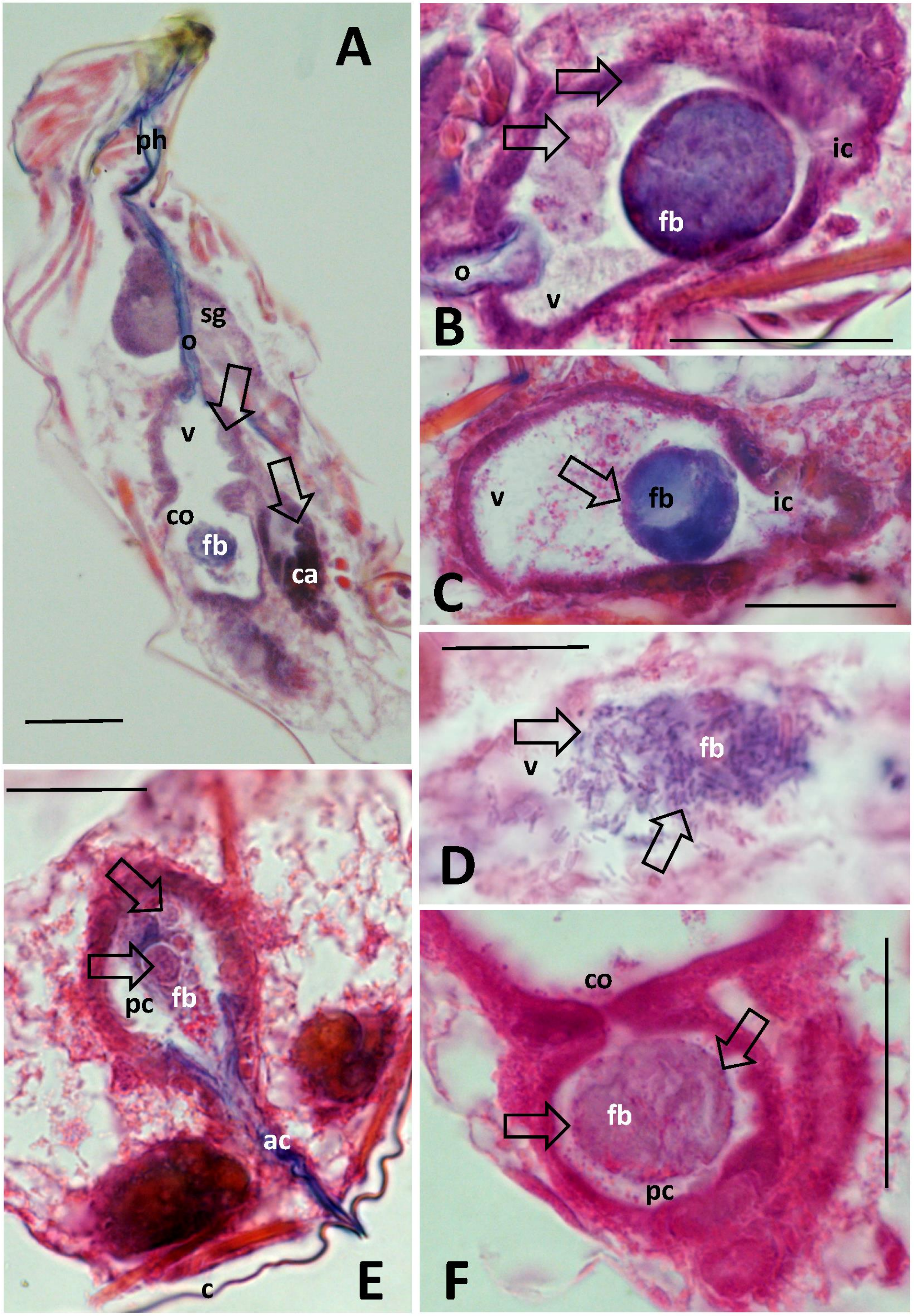
A
ph
sg
o
v
co
fb
ca
B
ic
fb
o
v
C
v
fb
ic
D
v
fb
E
pc
fb
ac
c
F
co
fb
pc

FIGURE S2 | The changes in abundance of selected OTUs in microbial profiles of *T. putrescentiae* among treatments. The columns are medians, and bars are interquartile ranges. The abundance of the sequences in standardized data set was transformed logarithmically (LOG2). The asterisk indicates significant differences (P < 0.05) from the control treatment in METASTATS.

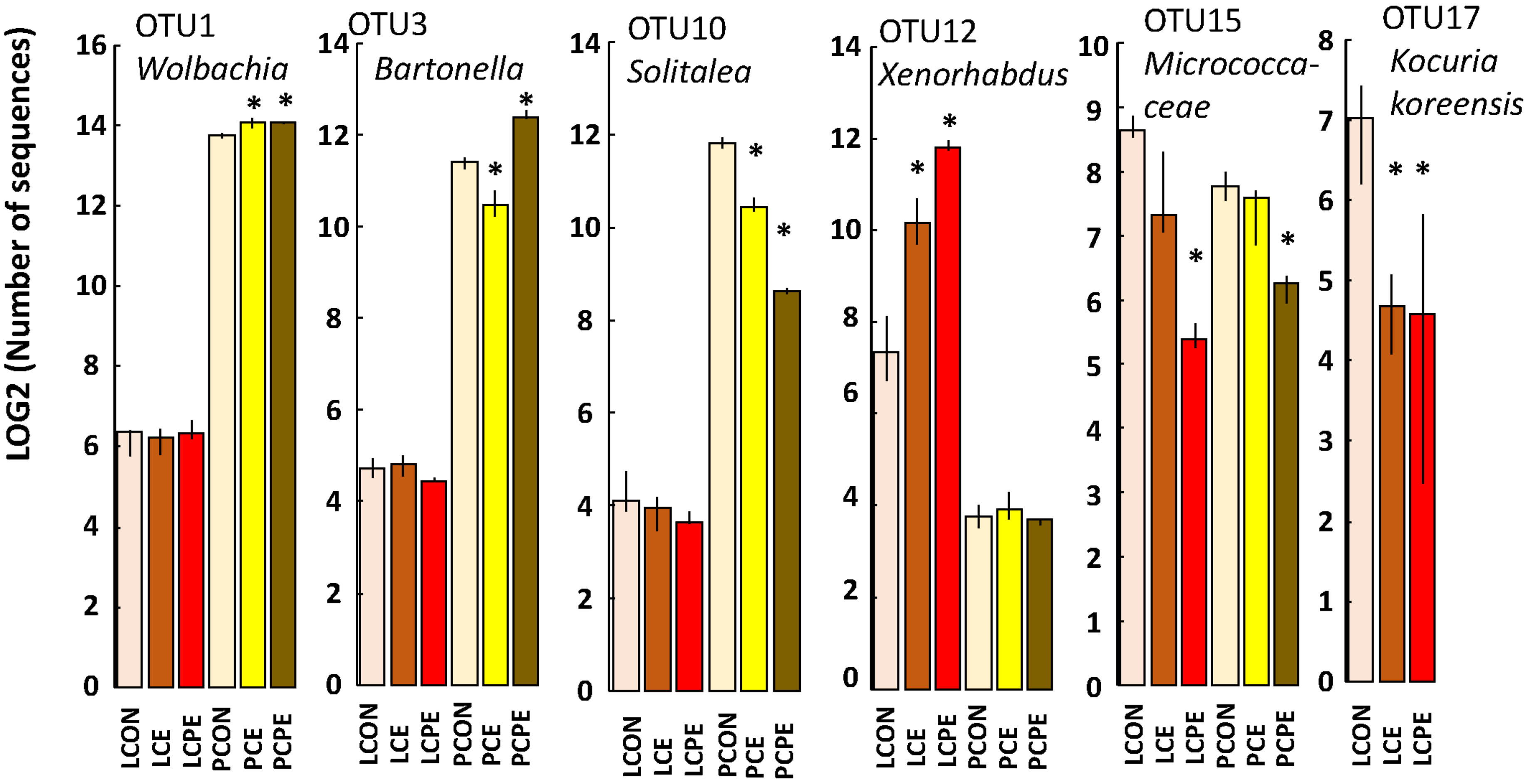

Supplement: Supplementary file 1 [file Data_Sheet_1.pdf]
